# Early Growth Response Gene 1 Benefits Autoimmune Disease by Promoting Regulatory T Cell Differentiation as a Regulator of Foxp3

**DOI:** 10.34133/research.0662

**Published:** 2025-04-15

**Authors:** Liu Yang, Xinyan Han, Mengxue Wang, Xiaojuan Zhang, Lupeng Wang, Nuo Xu, Hui Wu, Hailian Shi, Weidong Pan, Fei Huang, Xiaojun Wu

**Affiliations:** ^1^ Shanghai Key Laboratory of Compound Chinese Medicines, The Ministry of Education (MOE) Key Laboratory for Standardization of Chinese Medicines, The MOE Innovation Centre for Basic Medicine Research on Qi-Blood TCM Theories, Institute of Chinese Materia Medica, Shanghai University of Traditional Chinese Medicine, Shanghai, China.; ^2^ Central Laboratory, Shuguang Hospital Affiliated to Shanghai University of Traditional Chinese Medicine, Shanghai, China.; ^3^ Department of Neurology, Tangdu Hospital, Air Force Medical University, Xi’an, China.; ^4^ Department of Neurology, Shuguang Hospital Affiliated to Shanghai University of Traditional Chinese Medicine, Shanghai, China.

## Abstract

Foxp3^+^ regulatory T (T_reg_) cells, as one of the subtypes of CD4^+^ T cells, are the crucial gatekeeper in the pathogenesis of self-antigen reactive diseases. In this context, we demonstrated that the selective ablation of early growth response gene 1 (Egr-1) in CD4^+^ T cells exacerbated experimental autoimmune encephalomyelitis (EAE) in murine models. The absence of Egr-1 in CD4^+^ T cells, obtained from EAE mice and naïve CD4^+^ T cells, impeded the differentiation and influence of T_reg_. Importantly, in CD4^+^ T cells of multiple sclerosis patients, both Egr-1 and Foxp3 were found to decrease. Further studies showed that distinct from the classical Smad3 route, TGF-β could activate Egr-1 through the Raf–Erk signaling route to promote Foxp3 genetic modulation, thereby promoting T_reg_ cell differentiation and reducing EAE inflammation. A novel natural Egr-1 agonist, calycosin, was found to attenuate EAE progression by regulating the differentiation of T_reg_. Together, the above results indicate the value of Egr-1, as a novel Foxp3 transactivator, for the differentiation of T_reg_ cells in the development of self-antigen reactive diseases.

## Introduction

CD4^+^ T lymphocyte mediates many self-antigen reactive or inflammatory diseases, such as multiple sclerosis (MS), inflammatory bowel disease (IBD), rheumatoid arthritis (RA) [1], and so on. Immunologically, self-antigen reactive disease correlates with regulatory T (T_reg_) dysinfluence and enhanced T helper 1 (T_H_1) and T_H_17 responses [2]. This immunological imbalance may stem from the compromised suppressive action of T_reg_ cells on impactor T effector (T_eff_). MS is typified by persistent inflammation, prevalent demyelination, and axonal deterioration [3]. Experimental self-antigen reactive encephalomyelitis (EAE) serves as a canonical animal model for MS [4]. In adoptive transfer studies, T_reg_ cells have demonstrated the potential to impede the emergence of chronic EAE in recipient mice, suggesting a protective impact for T_reg_ cells in the context of MS [5,6]. Furthermore, many MS patients have decreased forkhead box protein 3 (Foxp3) manifestation in T_reg_ cells, showing weakened suppressive influence [7]. Foxp3-expressing natural T_reg_ cells, as one of the best-studied subtypes of CD4^+^ regulative T cells, play a crucial impact during MS progression [8]. The pathogenesis of MS/IBD is apparently influenced by T_H_17 cells, which secrete the pro-inflammatory cytokine interleukin-17A (IL-17A). T_reg_ cells counteract inflammation and modulate immune response by releasing the anti-inflammatory cytokine IL-10 [9]. Augmenting the ratio of T_reg_ cells is broadly regarded as an efficacious strategy for MS/IBD treatment. Consequently, T_reg_ cell therapy holds promise as a valuable tool for managing self-antigen reactive disorders.

CD4^+^ T cells can be partitioned into discrete groupings predicated on their observable traits and operative properties, with each faction bearing the onus for a specific immune reaction. Thus, the metamorphosis of inexperienced CD4^+^ T cells into a particular subdivision represents an indispensable course of action for upholding immune system poise. This progression is regulated by explicit genetic modulators that oversee the expression of singular surface receptors and cytokines. As an example, T-box genetic modulation modulator (T-bet) masterminds T_H_1 transmutation, thereby encouraging interferon-γ (IFN-γ) fabrication; conversely, GATA-connection protein 3 (GATA-3) commands T_H_2 evolution, eventuating in IL-4 synthesis [10]. The T_H_17-specific retinoic acid receptor-related orphan receptor γt (RORγt) synchronizes the exhibition of IL-17 [11]. T_H_1 and T_H_17 cells cooperate to nurture inflammation and are pivotal for the commencement of MS/EAE [12]. In contradistinction, T_reg_ cells tame the force and extent of the reaction and abet forbearance institution [13]. Deteriorated evolution or capability of T_reg_ cells spawn severe self-antigen reactive maladies such as MS. Therefore, schemes that amplify T_reg_ cell differentiation or invigorate their suppressive competencies materialize as promising therapeutic involvements for MS/EAE [14,15].

The Foxp3 genetic modulation modulator holds the position of the master regulator for T_reg_ cells [16]. Basal manifestation of Foxp3 hinges on the regulative interplay of numerous T cell receptor (TCR)-induced genetic modulation modulators [17–19]. Nevertheless, achieving maximal and sustained manifestation of Foxp3 demands additional stimulation from cytokines like IL-2 and TGF-β [20]. Consequently, the differentiation and functional regulation of T_reg_ cells are predominantly reliant on the modulation of their genetic modulation modulator Foxp3. While certain clinical therapies for MS can promote T_reg_ cell differentiation and function, the complete functional mechanism of T_reg_ cells in MS remains partially understood, and its impact is often regulated by many genetic modulation modulators and affected by the inflammatory environment in which it is located. In addition, the immune regulation network centered on T_reg_ is extremely complex and huge. In this way, it will be more helpful to understand the mechanism of MS and other related self-antigen reactive diseases by continuing the in-depth study of T_reg_ and its related regulative modulators, so as to better the clinical diagnosis and therapy.

A constituent of the Cys^2^ His^2^-type zinc finger genetic modulation modulator family, early growth response gene 1 (Egr-1) possesses the ability to attach to gene stimulator regions. This genetic modulation modulator is engaged in multiple cellular procedures and assumes a crucial part in the immune system (including a variety of physiologically relevant and immune impactor genes) containing the specific sequence of 5′-CGCCC (A/C) CGC-3′ and directly regulates its manifestation [21]. The manifestation of Egr-1 is strictly regulated by its upstream signaling route [22].

It is well established that mitogen-activated protein kinase kinase (Mek)–extracellular signal-regulated kinase (Erk) serves as a classical signaling route governing the genetic modulational manifestation of Egr-1. An inverse correlation between Egr-1 manifestation in CD4^+^ T cells and the severity of EAE in mice is shown. Additionally, lower Egr-1 manifestation levels were found in the blood of MS patients when in contrast with their healthy counterparts, with a parallel observation in mice with IBD. Notably, Egr-1 activation contributed to T_reg_ cell differentiation and alleviation of EAE inflammation through its connection to the Foxp3 stimulator region. Moreover, TGF-β modulated Foxp3 manifestation via the Raf/Mek/Erk signaling route that targets Egr-1 activation, contrasting with the classical Smad3 route. A newly identified natural Egr-1 agonist, calycosin (CAL), displayed potential in attenuating EAE progression by regulating T_reg_ differentiation. Collectively, these findings unveil a previously undescribed mechanism underpinning T_reg_ cell differentiation and development in MS/EAE and may present a fresh molecular target for the curative intervention of self-antigen reactive diseases.

## Results

### Egr-1 deficiency in CD4^+^ T cells aggravated EAE in mice

**Fig. 1.**
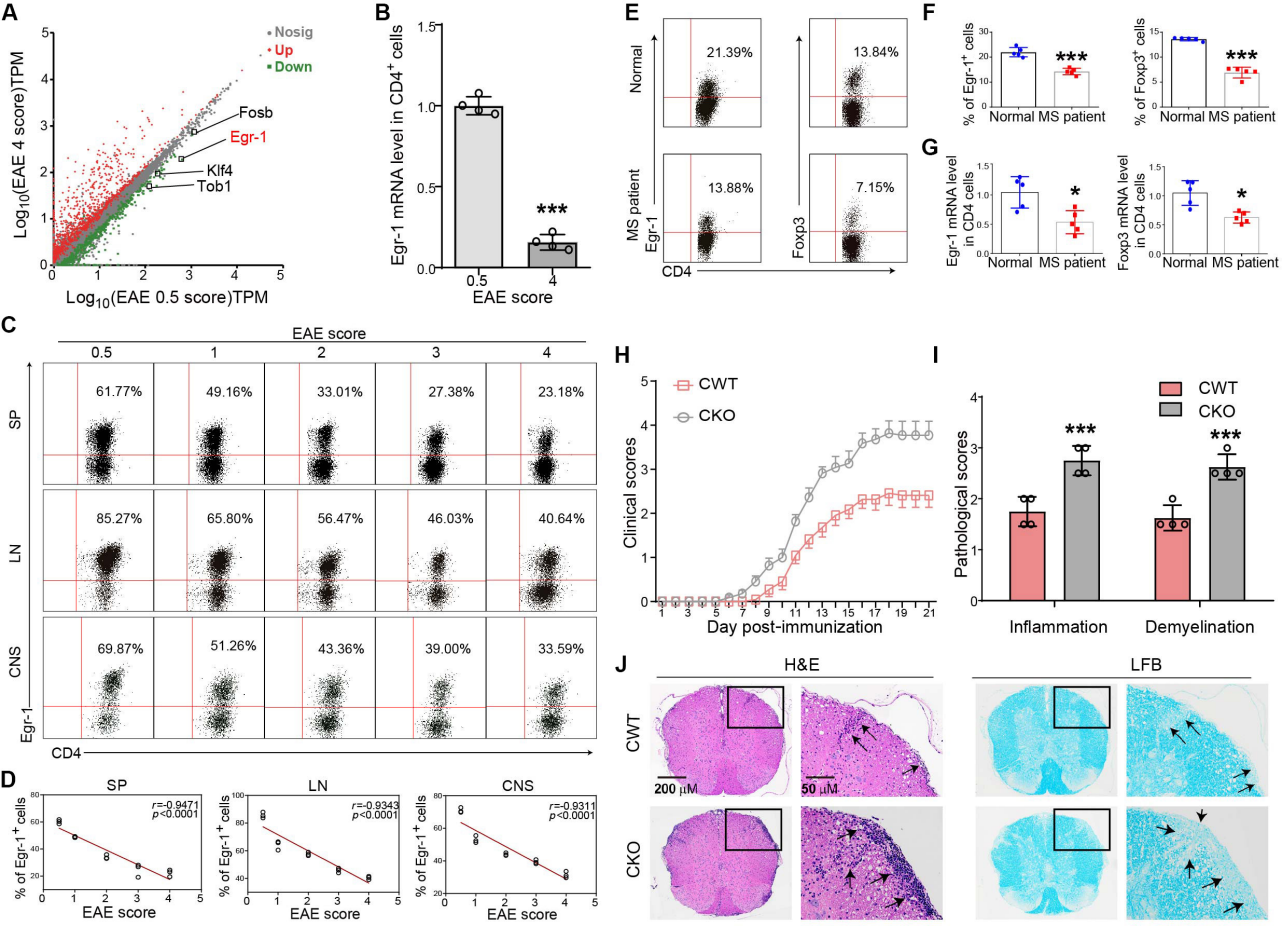
Expression of Egr-1 in CD4^+^ T cells was inversely related to the severity of EAE in mice. (A) RNA sequencing screened genes differentially expressed in CD4^+^ T cells in the spleen of EAE mice with mild disease (neurofunctional score of 0.5) and severe disease (neurofunctional score of 4). (B) Comparison of mRNA manifestation of Egr-1 in CD4^+^ T cells of EAE mice with mild disease (neurofunctional score of 0.5) and severe disease (neural functional score of 4) by qPCR. *n* = 4. (C and D) Egr-1 manifestation in EAE mice with different scores and correlation analysis of Egr-1 manifestation in CD4^+^ T cells with EAE scores. *n* = 4. SP, spleen; LN, lymph nodes. (E to G) CD4^+^ T cells in PBMCs of treatment-naïve MS patients and healthy donors. Afterward, the cells were stimulated with anti-CD3 and anti-CD28 in vitro for 24 h, and the manifestation of Egr-1 and Foxp3 was analyzed. *n* = 5. (H) EAE was induced in CWT and CKO mice by immunizing with MOG_33–55_ peptide and neurobehavioral defects. *n* = 11. (I and J) H&E and LFB staining pathological assessment of spinal cords from CWT and CKO EAE mice on day 21 post-immunization. *n* = 4. Data are expressed as mean ± SD. *P < 0.05; **P < 0.01; ***P < 0.001.

With the aim of identifying genes uniquely expressed in CD4^+^ T cells that contribute to the development of EAE in mice, we conducted a comparative transcriptomic analysis of CD4^+^ T cells from EAE mice exhibiting mild disease symptoms (neurobehavioral score of 0.5) and severe disease (neurobehavioral score of 4) by RNA sequencing. RNA sequencing identified differentially expressed genes (DEGs) were shown in Fig. 1A. We mainly focused on the top 100 differential genes with high manifestation levels in CD4^+^ T cells. Among the 100 genes, FBJ osteosarcoma oncogene B (Fosb), Egr-1, kruppel-like modulator 4 (Klf4), and transducer of ErbB-2.1 (Tob1) are the genetic modulation modulators, and Egr-1 was the mostly altered one (Fig. 1A). Quantitative polymerase chain reaction (qPCR) analysis corroborated the apparently diminished mRNA manifestation of Egr-1 in CD4^+^ T cells of EAE mice exhibiting severe symptoms (Fig. 1B; *P* < 0.001). Additionally, an inverse correlation was observed between the ratio of Egr-1-positive cells in CD4^+^ T cells and the escalation of neurobehavioral scores, regardless of their location within the spleen, lymph nodes, or central nervous system (CNS) of EAE mice (Fig. 1C and D; *P* < 0.001). Collectively, these findings indicate that Egr-1 manifestation in CD4^+^ T cells is inversely associated with the severity of EAE.

To investigate Egr-1 and Foxp3 manifestation in CD4^+^ T cells from MS patients, we segregated CD4^+^ T cells from the peripheral blood mononuclear cells (PBMCs) of treatment-naïve MS patients. The results demonstrated a substantial reduction in the ratio of Egr-1- and Foxp3-positive CD4^+^ T cells in MS patients (Fig. 1E and F; *P* < 0.001). Moreover, at the mRNA level, the manifestations of Egr-1 and Foxp3 were diminished in CD4^+^ T cells from MS patients when compared to healthy controls (Fig. 1G; *P* < 0.05). These discoveries intimated that Egr-1 might play a kindred part in MS patients as witnessed in the murine model. Moreover, in mice with dextran sulfate sodium salt (DSS)-induced colitis, diminished manifestation of Egr-1 and Foxp3 was discerned in colon tissue (Fig. S1F; *P* < 0.01 and *P* < 0.001). Notedly, a substantial diminution in the rate of CD4^+^ Egr-1^+^ cells was perceptible in the spleen and lymph nodes (Fig. S1F; *P* < 0.001). Collectively, these results indicated that the down-regulation of CD4^+^ Egr-1 may serve as a contributing modulator in self-antigen reactive disease development.

To unravel the specific impact of Egr-1 in CD4^+^ T cells during the pathogenesis of EAE, we induced EAE in conditional wild-type (CWT) (Egr-1^f/f^) mice and conditional knockout (CKO) (Egr-1^f/f^ CD4-cre^+^) mice born in the same litters. As shown in Fig. 1H, the onset of EAE in CKO mice was apparently earlier than that in CWT mice (Table S1; *P* < 0.001), and the peak score was higher (Table S1; *P* < 0.01). Moreover, on day 21, EAE activation in CKO mice resulted in more inflammatory infiltration and demyelination in spinal cord than that in CWT mice, particularly in white matter (Fig. 1I and J; *P* < 0.001). The aforementioned results show Egr-1-specific knockout in CD4^+^ T lymphocyte aggravated EAE in mice.

### Egr-1 deficiency in CD4^+^ T cells weakened the differentiation and function of T_reg_ cells

**Fig. 2.**
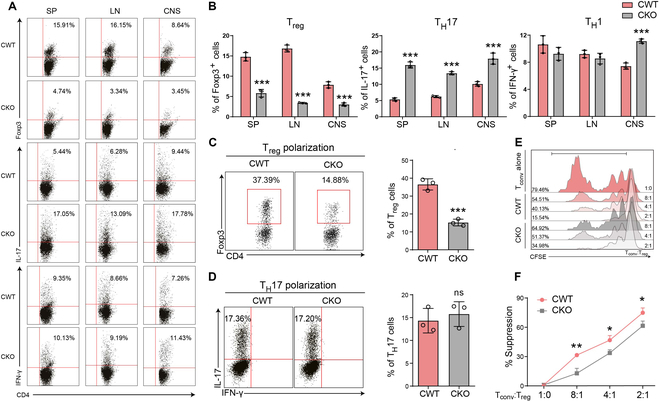
CD4^+^Egr-1 knockout regulated the subtypes of CD4^+^ T cell differentiation in EAE mice. (A) Proportions of T_reg_/T_H_17/T_H_1 cell subsets in spleen, lymph nodes, and CNS from CWT (Egr-1^f/f^) and CKO (Egr-1^f/f^ CD4-cre^+^) EAE mice. (B) Percentage of Foxp3-, IL-17-, and IFN-γ-positive cells. *n* = 4. (C and D) Representative flow cytometric chart and statistical bar graphs of the percentages of Foxp3- and IL-17-positive cells. *n* = 3. (E) Representative CFSE fluorescence flow cytometric chart of the inhibition of T_conv_ cells by T_reg_ polarized from naïve CD4^+^ T cells sorted from CWT and CKO mice and incubated for 3 d under T_reg_ polarization conditions. These cells were co-incubated with CFSE-labeled responder naïve CD4^+^ T cells. (F) Statistical analysis of the suppression rate of the T_reg_ from CWT and CKO mice on T_conv_ cells. Suppression % = (1 − CFSE fluorescence reduction percentage of T_conv_ cells) × 100%. *n* = 4. Data are expressed as mean ± SD. ns (not significant), *P* > 0.05; *P < 0.05; ***P < 0.001.

To further probe the impact of Egr-1 excision on CD4^+^ T lymphocyte differentiation in EAE murines, we initially discerned no substantial discrepancies in the rates of CD3/CD19 and CD4/CD8 in the spleen and lymph nodes of typical conditional CWT and CKO rodents (Fig. S2). This indicates that CD4 Egr-1 excision did not affect the rates of immune cells in hale murines (*P* > 0.05). After eliciting EAE in CWT and CKO mice, we gauged the ratios of CD4^+^ T and T_H_17/T_reg_/T_H_1 lymphocyte subtypes in the spleen, lymph nodes, and CNS. We witnessed a clear disequilibrium in the T_reg_/T_H_17 rate of CD4^+^ T cells in CKO EAE murines contrasted with CWT EAE murines (Fig. 2A and B). Notably, the rate of T_reg_ cells was considerably dwindled (*P* < 0.001), while the rate of T_H_17 cells was augmented (*P* < 0.001). In contradistinction, the rate of T_H_1 cells in CKO EAE murines stayed predominantly unaltered in the spleen and lymph nodes but escalated in the CNS (*P* < 0.001).

To examine the direct impact of Egr-1 on T_H_17 and T_reg_ cell differentiation in vitro, we incubated naïve CD4^+^ T cells from CWT Egr-1 CKO mice under T_H_17 or T_reg_ polarization conditions. Our findings revealed that Egr-1 knockout apparently impaired the ability of T_reg_ differentiation, while having no impact on their capacity to differentiate into T_H_17 cells (Fig. 2C and D; *P* < 0.001 and *P* > 0.05, respectively). We further investigated whether Egr-1 deficiency weakened the suppressive activity of T_reg_ cells. In Fig. 2E and F, the suppression ratio of T_reg_ cells differentiated from CD4^+^ T cells in CKO mice against responder cells was lower than that from CWT mice (*P* < 0.05 and *P* < 0.01). The above results demonstrate that Egr-1 knockout in CD4^+^ T cells directly impairs T_reg_ cell differentiation and suppressive function.

### Adoptive transfer of CD4^+^ T cells from CKO EAE mice led to the aggravation of EAE in WT mice

**Fig. 3.**
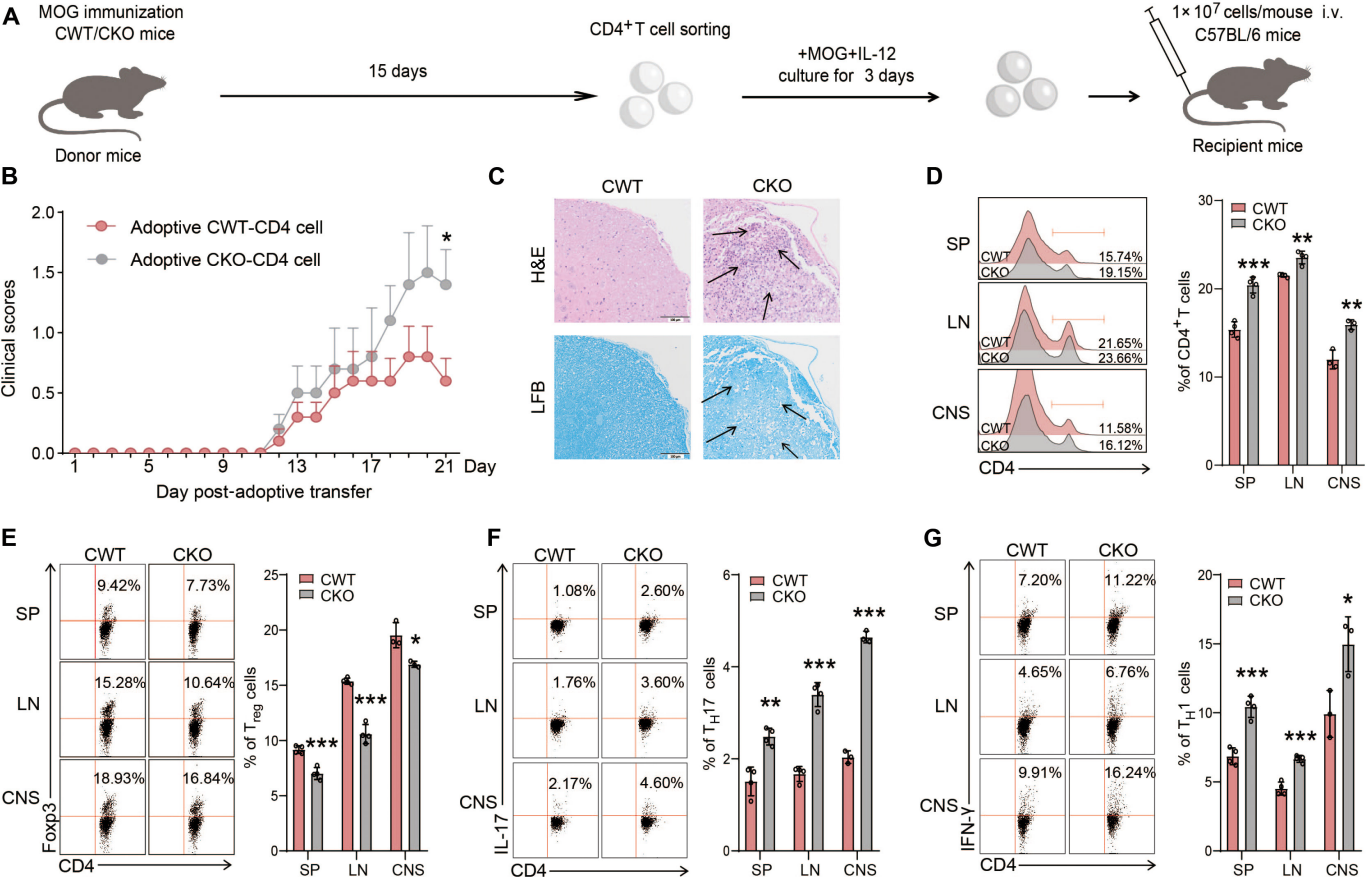
Adoptive transfer of CD4^+^ T cells of CKO EAE mice led to the aggravation of EAE in WT mice. (A) Experimental scheme of the adoptive transfer. CKO and CWT mice were immunized with MOG_35–55_ for 15 d. Then, CD4^+^ T cells from lymph node cells were incubated with MOG_35–55_ (20 μg/ml) and IL-12 (0.5 ng/ml) for 3 d. The treated cells (1 × 10^7^ cells/mouse) were then injected into WT C56BL/6 recipient mice through the tail vein. (B) Clinical score. *n* = 6. (C) H&E and LFB staining and inflammatory infiltration and demyelination scores in the spinal cord. (D) Proportion of CD4^+^ T cells in spleen, lymph nodes, and CNS of mice. *n* = 3 to 4. (E) Proportion of CD4^+^Foxp3^+^T_reg_ cells in CNS, spleen, and lymph nodes of mice. (F) Proportion of CD4^+^IL-17^+^T_H_17 cells in CNS, spleen, and lymph nodes of mice. (G) Proportion of CD4^+^IFN-γ^+^T_H_1 cells in CNS, spleen, and lymph nodes of mice. Data are expressed as mean ± SD. *P < 0.05; **P < 0.01; ***P < 0.001 by Student’s *t* test.

To further understand the impact of CD4^+^ T lymphocyte Egr-1 during EAE, we compared the severity of EAE progression by transferring CD4^+^ T cells from CKO and CWT EAE mice into WT recipient mice. Briefly, we induced EAE in CKO and CWT mice for 15 d and then isolated CD4^+^ T cells from their lymph node cells. These cells were incubated with MOG_35–55_ (20 μg/ml) and IL-12 (0.5 ng/ml) for 3 d before being injected into WT recipient mice (1 × 10^7^ cells/mouse) through the tail vein (Fig. 3A). Our results demonstrated that mice receiving CKO CD4^+^ T cells showed an apparently higher clinical score at day 21 compared to mice receiving CWT CD4^+^ T cells (Fig. 3B; *P* < 0.05). Moreover, the transfusion of CKO CD4^+^ T cells precipitated a noteworthy escalation in the quotient of CD4^+^ T cells in both cardinal and peripheral murine models (Fig. 3C and D; *P* < 0.01 and *P* < 0.001), accompanied by a discernible depletion in the rate of T_reg_ cells and a substantial amplification in the rate of T_H_17 and T_H_1 cells (Fig. 3E to G; *P* < 0.05, *P* < 0.01, and *P* < 0.001). Jointly, these results render supplemental substantiation for the office of Egr-1 in orchestrating the differentiation and efficacy of T_reg_ during the pathogenic processes of inflaming incursion and demyelination within the context of EAE.

### Egr-1 regulated the differentiation of T_reg_ cells as a transactivator of Foxp3

**Fig. 4.**
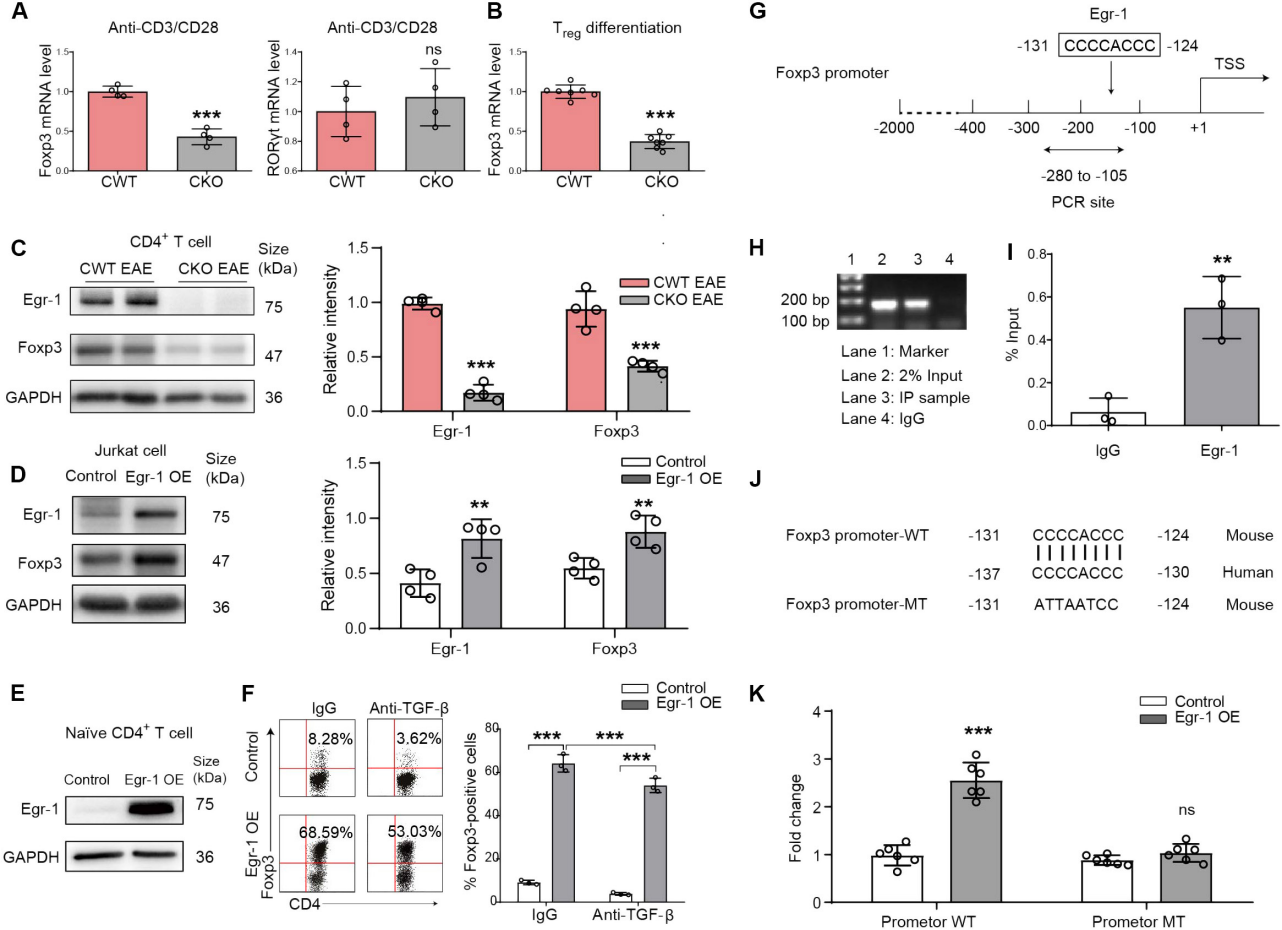
Egr-1 induced the manifestation of Foxp3 by connection to its promoter site. (A) mRNA manifestation of Foxp3 and RORγt in CD4^+^ T cells sorted from the spleens of CWT and CKO mice, after stimulation with anti-CD3 (5 μg/ml) plus anti-CD28 (2 μg/ml) for 6 h. (B) mRNA manifestation of Foxp3 under T_reg_ polarization condition. (C) Protein manifestation of Egr-1 and Foxp3 in CD4^+^ T cells of CWT and CKO EAE mice. (D) Protein manifestation of Egr-1 and Foxp3 in Jurkat cells transfected with PCB6-Egr-1 and PCB6 empty plasmids. (E) Egr-1 protein manifestation in naïve CD4^+^ T cells sorted from normal mice and transfected with PCB6-Egr-1 plasmid and PCB6 control plasmid. (F) Expression of Foxp3 in naïve CD4^+^ T cells stimulated with TCR under neutral conditions and transfected with either PCB6-Egr-1 plasmid or PCB6 control plasmid in the presence of anti-TGF-β or control IgG. *n* = 4. (G) Schematic diagram depicting and the putative Egr-1 connection site (−131 bp to −124 bp). (H and I) ChIP analysis performed using a negative control immunoglobulin G (IgG) or anti-Egr-1 antibody in CD4^+^ T cells of EAE mice. (J) Schematic diagram depicting the sequences in the WT and mutant (MT) Foxp3 stimulator. (K) Luciferase activity analysis in EL4 T cells transfected with Foxp3 Promoter-WT plasmid and Foxp3 Promoter-MT plasmid after overexpressing Egr-1. *n* = 6. Data are expressed as mean ± SD. ns, *P* > 0.05; ***P < 0.001.

Since Foxp3 controls the differentiation of T_reg_, we next examined the influence of Egr-1 in the regulation of Foxp3. Our data unveiled that Foxp3’s mRNA articulation was appreciably stifled in splenic CD4^+^ T cells harvested from Egr-1-deficient murines upon provocation with anti-CD3 and anti-CD28, while RORγT’s mRNA articulation persisted unperturbed (Fig. 4A; *P* < 0.001 and *P* > 0.05). In concordance, upon incubation under T_reg_-fomenting milieu, purified naïve CD4^+^ T cells exhibited a discernible enfeeblement in Foxp3’s mRNA articulation within Egr-1-deficient counterparts (Fig. 4B; *P* < 0.001). In EAE-induced mice, the protein manifestations of Egr-1 and Foxp3 were both decreased from CKO mice (Fig. 4C; *P* < 0.001). Conversely, overmanifestation of Egr-1 increased Foxp3 manifestation in the Jurkat T lymphocyte line (Fig. 4D; *P* < 0.01). To further investigate the impact of Egr-1 on Foxp3 manifestation in CD4^+^ T cells, we transfected naïve CD4^+^ T cells with the PCB6-Egr1 plasmid, which induces the overmanifestation of Egr-1 protein (Fig. 4E). Upon transfection of sorted naïve CD4^+^ T cells from normal mice with the PCB6-Egr1 plasmid under neutral conditions, we observed a clear growth in the percentage of Foxp3^+^ cells as compared to those transfected with the control (PCB6) plasmid (Fig. 4F; *P* < 0.001). Egr-1-mediated Foxp3 activation was still observed even when TGF-β was neutralized by immunoglobulins (Fig. 4F; *P* < 0.001), suggesting that Egr-1 inherently promotes Foxp3 manifestation. However, when TGF-β was neutralized, the Foxp3 manifestation in CD4^+^ T cells was markedly reduced (*P* < 0.001). These outcomes imply that Egr-1 may govern T_reg_ cell differentiation through the direct regulation of Foxp3 and TGF-β manifestation.

To elucidate the specific mechanism by which the genetic modulation modulator Egr-1 regulates Foxp3 manifestation, we utilized the JASPAR and UCSC databases to identify a potential Egr-1 connection site (Fig. 4G) in the Foxp3 promoter region [−131 to −124 base pairs (bp)]. We subsequently substantiated this liaison locus employing chromatin immunoprecipitation (ChIP)-PCR. As evinced in Fig. 4H, a momentous enrichment of the conjectural Egr-1-liaison locus (−131 to −124 bp) was discerned post-immunoprecipitation with an anti-Egr-1 antibody, while null band was described upon immunoprecipitation with a negative control IgG antibody. ChIP-qPCR outcomes farther corroborated that Egr-1 protein/Foxp3 gene complexes were dragooned by the anti-Egr-1 antibody in CD4^+^ cells of EAE mice (Fig. 4I, *P* < 0.01). To instate the pinpoint locale of the Egr-1-liaison locus within the Foxp3 promoter sequence and to authenticate the functional nexus between the Egr-1-liaison locus and Foxp3 promoter vim, we wielded ancillary experiments. As illustrated in Fig. 4J, we selected base sequence that 2,000 bp upstream of Foxp3 genetic modulation start site and constructed a luciferase reporter gene plasmid containing Foxp3 promoter sequence (referred to as Promoter-WT) and designed a reporter gene plasmid with mutation sequence (referred to as Promoter-MT) for the connection sequence (−131 bp to −124 bp). In addition, the Foxp3 promoter connection sequences both of mouse and human were compared and found to be highly conservative (Fig. 4J). After the Egr-1 was overexpressed, the Foxp3 Promoter-WT plasmid and Foxp3 Promoter-MT plasmid were transfected into the EL4 T cells. It turns out that the overmanifestation of Egr-1 caused a strong increase for the Foxp3 stimulator reporter gene activity. Egr-1 overmanifestation apparently enhanced the activity of WT Foxp3 stimulator reporter gene but did not affect the activity of the mutated Foxp3 stimulator reporter gene (Fig. 4K). Collectively, these findings suggest that Egr-1 directly regulates Foxp3 genetic modulation in CD4^+^ T cells, thereby influencing its protein levels.

### TGF-β modulated T_reg_ differentiation by activating Egr-1 through Raf/Mek/Erk, distinct from the classical Smad3 pathway

**Fig. 5.**
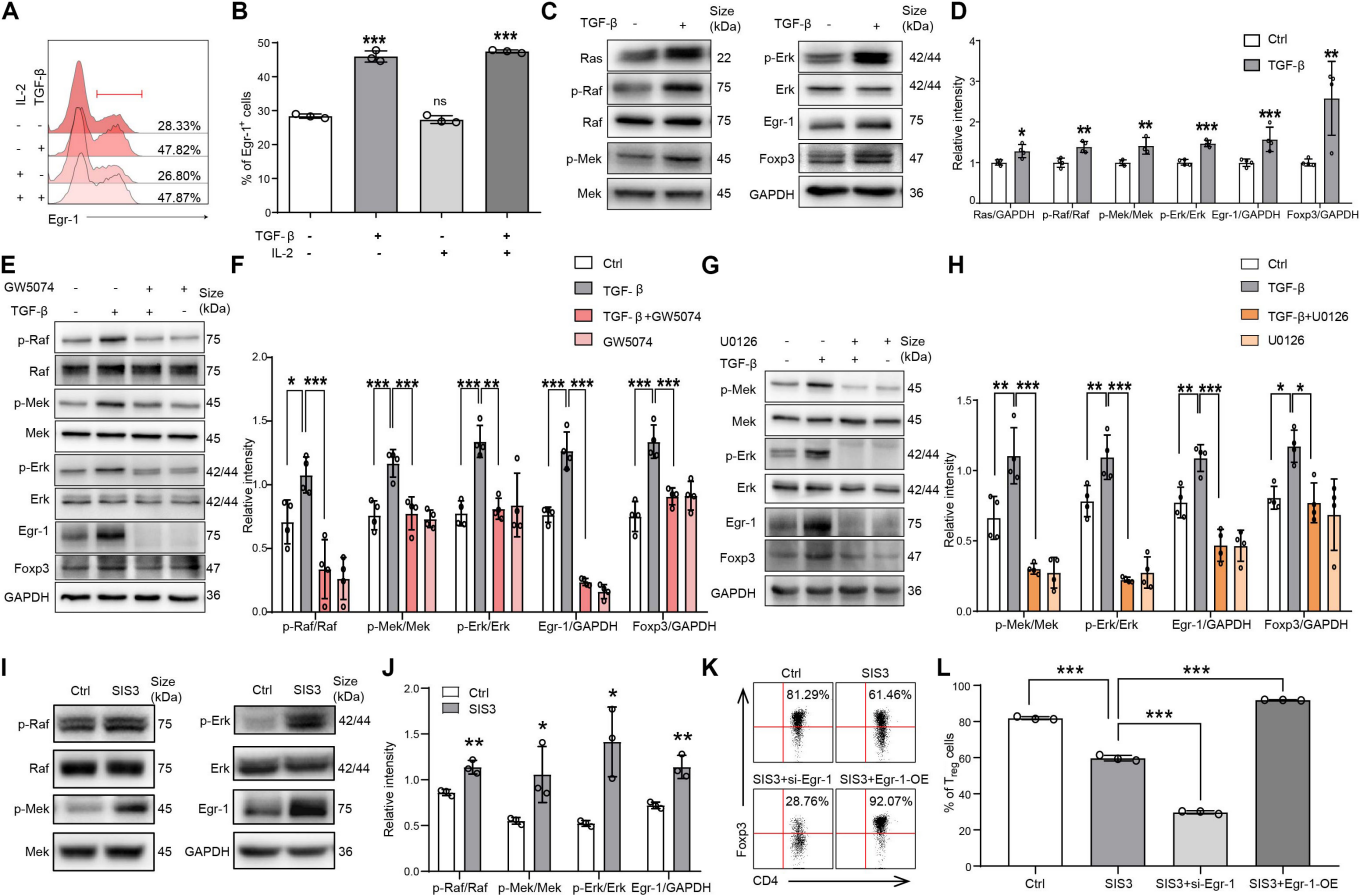
TGF-β regulated the manifestation of Egr-1 in CD4^+^ T cells through the Ras/Raf/Mek/Erk signaling route. (A) Representative flow cytometric chart of Egr-1 manifestation in naïve CD4^+^ T cells stimulated with TCR in the absence or presence of TGF-β and IL-2 for 24 h. (B) Statistical analysis of the percentage of Egr-1-positive cells. (C and D) Protein manifestation stimulated with TGF-β. (E and F) Protein manifestation after pretreatment with GW5074 (inhibitor of Raf, 10 μM). (G and H) Protein manifestation after pretreatment with U0126 (inhibitor of Erk and Mek, 10 μM). *n* = 4. (I and J) Expression of Raf/Mek/Erk/Egr-1 protein after adding SIS3 (1 μM) during T_reg_ differentiation. (K and L) Proportion of T_reg_ cell differentiation. Data are expressed as mean ± SD. *P < 0.05; **P < 0.01; ***P < 0.001.

To further examine the regulative modulators upstream of Egr-1 during T_reg_ differentiation, we initially investigated the impact of TGF-β and IL-2 on Egr-1 manifestation in CD4^+^ T cells. As depicted in Fig. 5A and B, during T_reg_ cell polarization, TGF-β apparently enhanced Egr-1 manifestation, while IL-2 had no apparent impact on Egr-1 manifestation levels (*P* < 0.001 and *P* > 0.05). In addition, the Egr-1 activation level was comparable between TGF-β alone stimulation and TGF-β plus IL-2 costimulation. Further studies found that TGF-β activated Ras and then accelerated the phosphorylation of Raf, Mek, and Erk as well as Egr-1 in Jurkat cells (Fig. 5C and D; *P* < 0.05, *P* < 0.01, and *P* < 0.001). Moreover, to examine whether TGF-β activated Egr-1 through the Raf/Mek/Erk route, the Raf and Erk (Mek) inhibitors, GW5074 and U1026, were used, respectively. As shown in Fig. 5E and F, GW5074 reversed the activation of TGF-β on Raf, Mek, Erk, Egr-1, and Foxp3 (*P* < 0.05, *P* < 0.01, and *P* < 0.001). When U1026 was added, the impact of TGF-β in activating Mek, Erk, Egr-1, and Foxp3 disappeared (Fig. 5G and H; *P* < 0.05, *P* < 0.01, and *P* < 0.001). SIS3, a Smad3 inhibitor, was added during T_reg_ differentiation, and the results showed that inhibiting Smad3 resulted in compensatory up-regulation of the Raf/Mek/Erk/Egr-1 route (Fig. 5I and J). Flow cytometric evaluation revealed that SIS3 efficiently impeded the ratio of T_reg_ cell differentiation, as anticipated. Simultaneously, the interference of Egr-1 decreased T_reg_ cell differentiation to a lesser extent. However, Egr-1 overmanifestation mitigated the reduction in T_reg_ cell ratio caused by SIS3, suggesting a possible rescue impact (Fig. 5K and L; *P* < 0.001). The results suggest that distinct from the classical SIS3 route, the Raf-Egr-1 route can directly activate Foxp3 manifestation and promote T_reg_ differentiation.

### Egr-1 agonist CAL prevented EAE progression depending on Egr-1 of CD4^+^ T cells

**Fig. 6.**
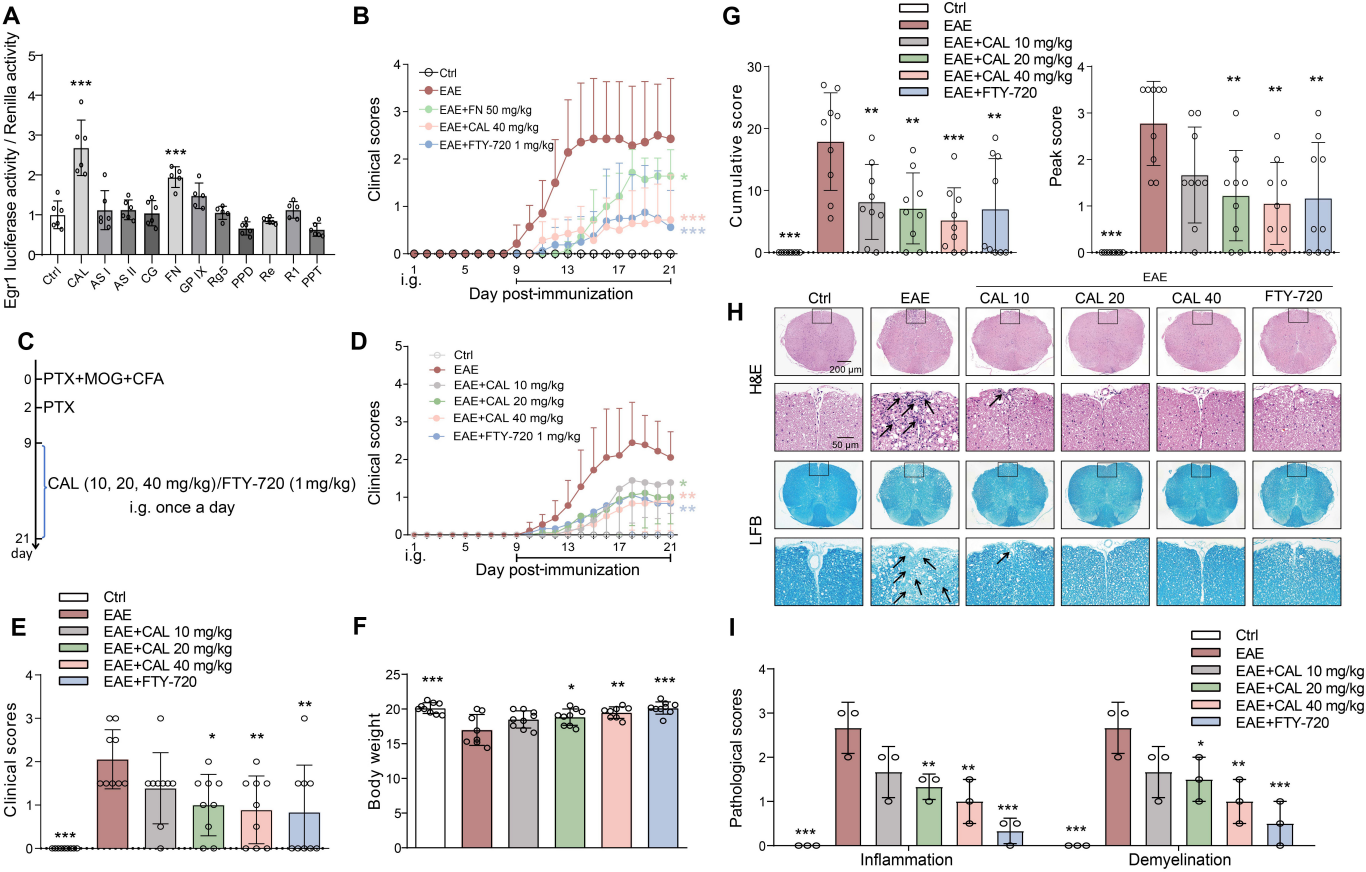
Egr-1 agonist CAL and FN had curative impact on EAE mice. (A) Screening of Egr-1 agonists by using ERE luciferase reporter gene. *n* = 5 to 6. (B) Daily neurobehavioral assessment of EAE mice treated with CAL, FN, FTY-720, or vehicle. *n* = 8. (C and D) Daily neurobehavioral assessment of EAE mice treated with CAL (10, 20, and 40 mg/kg), FTY-720, or vehicle. *n* = 9. (E) Neurobehavioral assessment. *n* = 9. (F) Body weight of EAE mice. *n* = 9. (G) Cumulative score and peak score. *n* = 9. (H and I) H&E and LFB staining and inflammatory infiltration and demyelination scores in the spinal cord of EAE mice. *n* = 3. Data are expressed as mean ± SD. *P < 0.05; **P < 0.01; ***P < 0.001.

To investigate the potential of Egr-1 as a novel curative target for MS, we first screened potential Egr-1 agonists using an ERE reporter gene assay. As depicted in Fig. 6A, CAL and formononetin (FN) substantially enhanced the ERE reporter luciferase activity in Jurkat cells (*P* < 0.001). Our subsequent findings demonstrated that Egr-1 agonists, CAL and FN, apparently attenuated the clinical scores of EAE mice (Fig. 6B; *P* < 0.05 and *P* < 0.001). Additionally, FTY-720, a US Food and Drug Administration (FDA)-approved drug for treating MS with T_reg_ activation impact, also notably reduced the clinical scores of EAE mice (Fig. 6B; *P* < 0.001). Given that CAL exhibited superior curative efficacy in EAE, we further explored its impact in EAE progression.

**Fig. 7.**
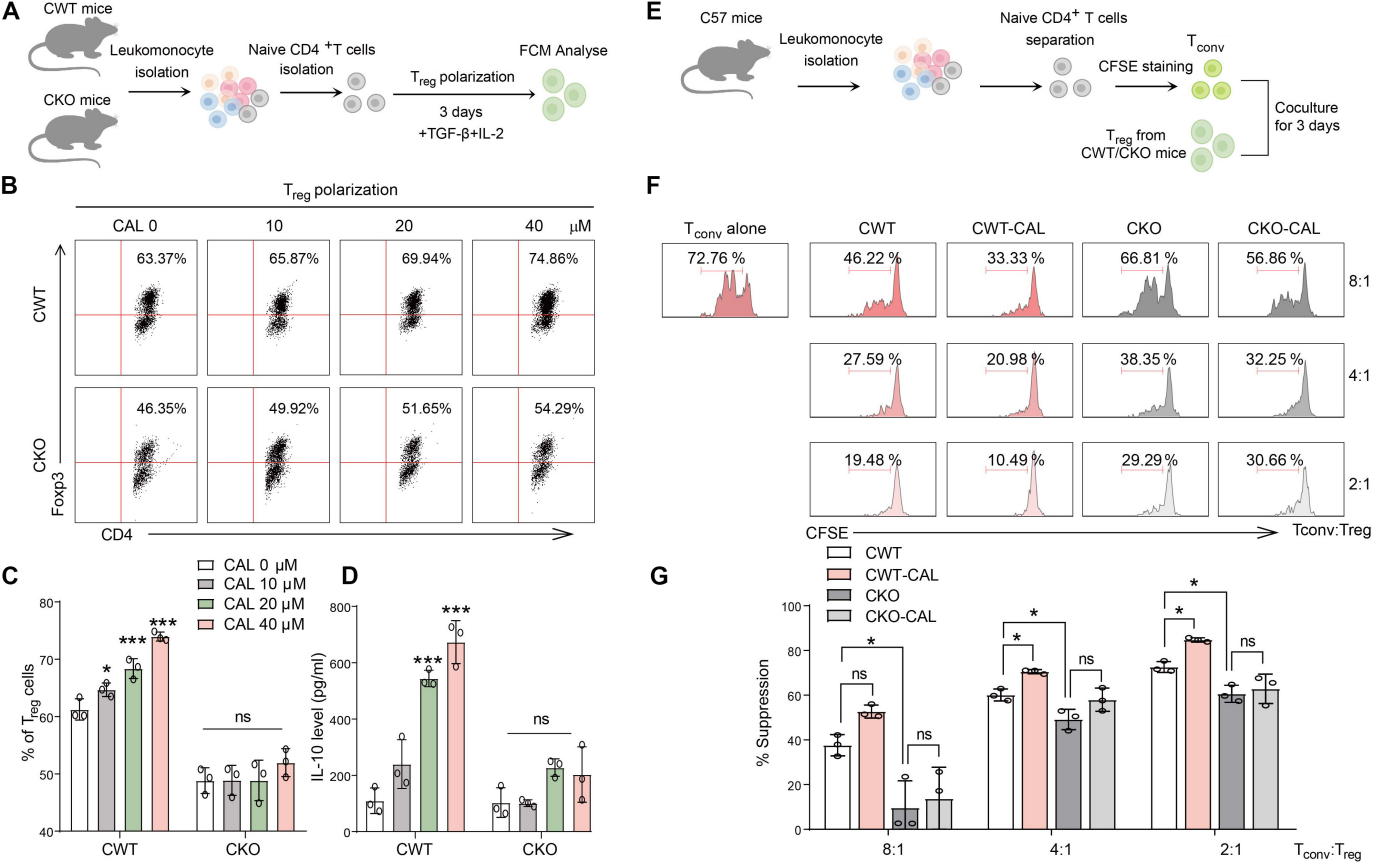
Egr-1 agonist CAL affected T_reg_ differentiation and influence through CD4^+^Egr-1. (A) Schematic diagram of T_reg_ differentiation. (B and C) Different concentrations of CAL were added during CWT/CKO T_reg_ differentiation, and the ratio of Foxp3^+^T_reg_ cells was detected. *n* = 3. (D) IL-10 secretion in T_reg_ cell incubation medium. *n* = 3. (E) Schematic diagram of T_reg_ immunosuppression. (F) Representative CFSE fluorescence flow cytometric chart of the inhibition of T_conv_ cells by T_reg_ polarized from naïve CD4^+^ T cells sorted from CWT and CKO mice and incubated with 40 μM CAL for 3 d under T_reg_ polarization conditions. These cells were co-incubated with CFSE-labeled responder naïve CD4^+^ T cells. (G) Statistical analysis of the suppression rate of the T_reg_ on T_conv_ cells. *n* = 3. Data are expressed as mean ± SD. ns, *P* > 0.05; *P < 0.05; **P < 0.01; ***P < 0.001.

In Fig. S3A, the ratio of CD4^+^EGFP^+^ cells in CAL-treated ERE-EGFP mice induced with EAE was increased apparently (*P* < 0.001). CAL increased the percentage of CD4^+^EGFP^+^ cells in naïve CD4^+^ T cells isolated from ERE-EGFP mouse lymph node under T_reg_ polarization condition (Fig. S3B; *P* < 0.01). These results implicated that CAL could activate Egr-1 manifestation both in vivo and in vitro. Therefore, to further confirm the curative impact of CAL on EAE, different doses of CAL (10, 20, and 40 mg/kg) were given by continuous intragastric administration starting from day 9 until day 21 after immunization (Fig. 6C). CAL demonstrated a dose-dependent amelioration of EAE (Fig. 6D to F; *P* < 0.05, *P* < 0.01, and *P* < 0.001) and apparently reduced peak and cumulative scores (Fig. 5G; *P* < 0.01 and *P* < 0.001). Furthermore, CAL notably mitigated spinal inflammatory infiltration and demyelination (Fig. 6H and I; *P* < 0.05, *P* < 0.01, and *P* < 0.001). CAL also diminished the ratio of activated CD4^+^ T cells, augmented the ratio of T_reg_ cells, decreased T_H_17 cell ratios in CNS mononuclear cells (MNCs), spleen, and lymph node cells, and reduced T_H_1 cell ratios in spleen cells (Fig. S4A to E; *P* < 0.05, *P* < 0.01, and *P* < 0.001). Additionally, CAL elevated IL-10 secretion levels in spleen cells, suppressed IL-17 and IFN-γ secretion, up-regulated Foxp3 mRNA manifestation, and down-regulated RORγt and T-bet mRNA manifestation (Fig. S4F; *P* < 0.05, *P* < 0.01, and *P* < 0.001). In MOG-restimulated EAE mice spleen cells, similar trends were observed (Fig. S5; *P* < 0.05, *P* < 0.01, and *P* < 0.001). Further, the impact of CAL on CWT/CKO T_reg_ cell differentiation was assessed. CAL dose-dependently increased CWT T_reg_ cell polarization and IL-10 secretion but had no marked impact on CKO T_reg_ cell differentiation or IL-10 levels (Fig. 7A to D; *P* > 0.05, *P* < 0.05, and *P* < 0.001). In the T_reg_ cell immunosuppression assay, CAL enhanced the immunosuppressive capacity of CWT T_reg_ but had no impact on CKO T_reg_’s immunosuppressive influence (Fig. 7E to G; *P* > 0.05 and *P* < 0.05). To uncover the impact of Egr-1 in the alleviative impact of CAL on EAE, CKO mice were administered with CAL after EAE activation. As a result, CAL manifested no curative impact on EAE in CD4^+^ T lymphocyte Egr-1 CKO mice (Fig. S6A and B; *P* > 0.05). The spinal cord demyelination and inflammatory infiltration of CKO EAE mice were not apparently improved after CAL treatment (Fig. S6C and D; *P* > 0.05). Furthermore, CAL had no impact on the ratio of CD4^+^ T lymphocyte and its subtypes in CKO EAE mice (Fig. S7; *P* > 0.05). Together, it suggested that CAL benefits the therapy of EAE in mice by activating Egr-1 of CD4^+^ T cells.

**Fig. 8.**
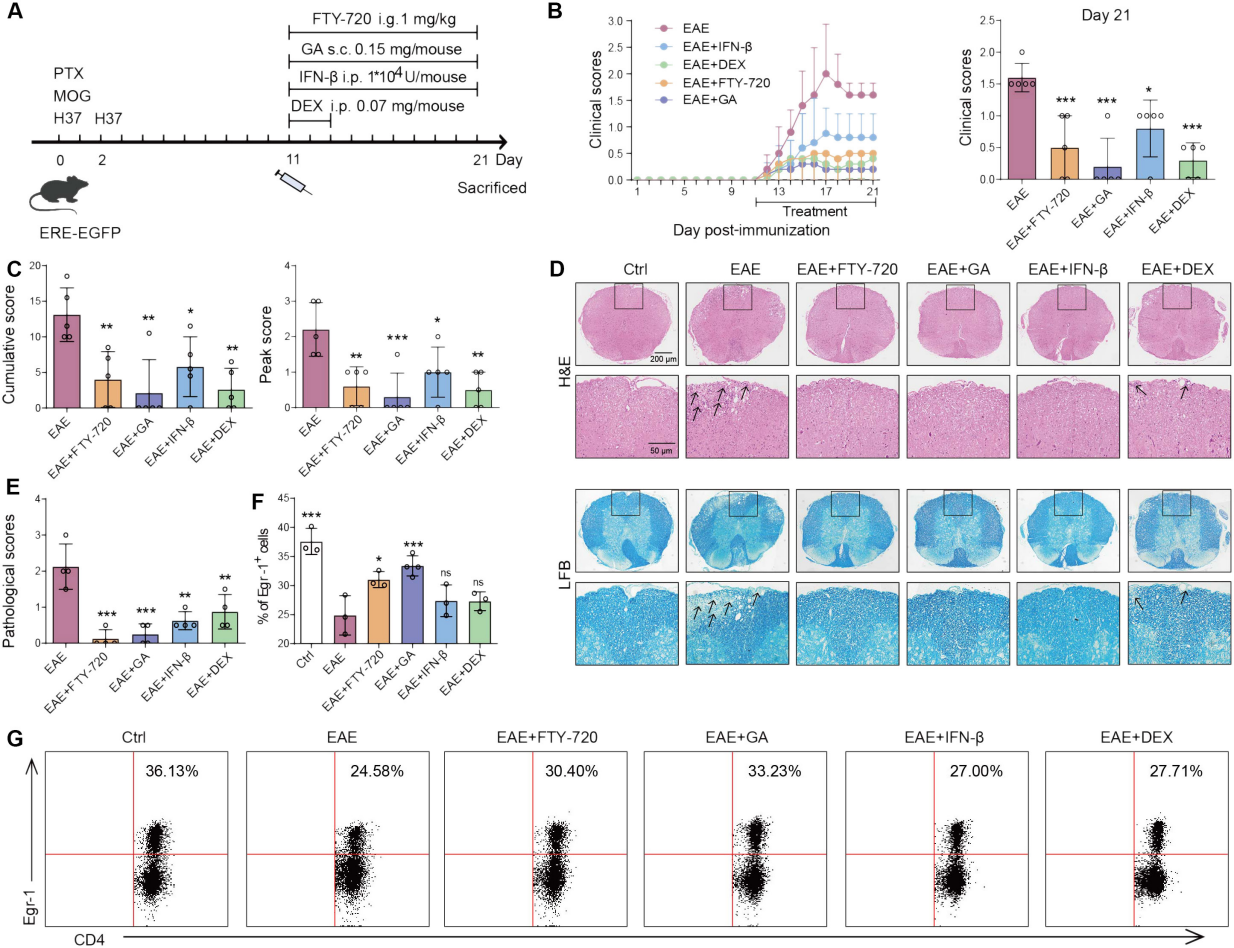
Clinical drugs for MS such as FTY-720 and GA could activate Egr-1 manifestation. (A) After 11 d of EAE activation in ERE-EGFP mice, FTY-720 (1 mg/kg), GA (0.15 mg/mouse), IFN-β (1 × 10^4^ U/mouse), and DEX (0.07 mg/mouse) were administered. *n* = 5. (B) Daily neurobehavioral assessment and score statistics. *n* = 5. (C) Cumulative score and peak score of EAE mice. *n* = 5. (D and E) H&E and LFB staining and inflammatory infiltration and demyelination scores in the spinal cord. *n* = 4. (F and G) Proportion of CD4^+^Egr-1^+^ cells in lymph nodes of EAE mice. *n* = 3. Data are expressed as mean ± SD. *P < 0.05; **P < 0.01; ***P < 0.001, versus EAE group.

To further verify the rationality of Egr-1 to be used as an impactive target in the treatment of MS, we examined the first-line drugs currently used in the clinical treatment of MS, including glatiramer acetate (GA; 0.15 mg/mouse), fingolimod (FTY-720; 1 mg/kg), IFN-β (1 × 10^4^ U/mouse), and dexamethasone (DEX; 0.07 mg/mouse) in terms of Egr-1 activation. As shown in Fig. 8A to C, all the 4 drugs reduced symptoms. CAL treatment also alleviated spinal cord inflammatory infiltration and demyelination in ERE-EGFP mice with EAE (Fig. 8D and E; *P* < 0.05, *P* < 0.01, and *P* < 0.001). Notably, only FTY-720 and GA apparently elevated the ratio of CD4^+^Egr-1^+^ cells in lymph node cells (Fig. 8F and G; *P* < 0.05 and *P* < 0.001). The findings imply that FTY-720 and GA may mitigate MS by activating Egr-1 in CD4^+^ T cells.

## Discussion

The current understanding is that autoreactive T cells, particularly CD4^+^ T cells, play a critical impact in initiating self-antigen reactive rejoinders partaking in the engenderment of self-antigen reactive diseases like MS. Consequently, our research primarily focused on CD4^+^ T cells to investigate the underlying mechanisms of MS/EAE pathogenesis. We first tried to tease out the potential functional genes in CD4^+^ T cells that get involved in the aggravation of MS by comparing the transcriptomes of CD4^+^ T cells from EAE mice with mild disease and severe disease. We found that Egr-1 was the greatest altered genetic modulation modulator among the top 100 differential genes with high manifestation levels in CD4^+^ T cells. Egr-1, a constituent of the Cys^2^ His^2^-type zinc finger genetic modulation modulator family that comprises Egr-1, Egr-2, Egr-3, and Egr-4, is swiftly stimulated in reaction to TCR signals and interfaces with the nuclear modulator of activated T cells (NFAT), partaking in CD4^+^ T lymphocyte differentiation. It has been established that the diminished Egr-1 manifestation in CD4^+^ T cells expedited the emergence of self-antigen reactive diabetes, while its overproduction deferred the inception of the disease, spotlighting Egr-1’s crucial influence in self-antigen reactive diseases.

In the present investigation, we ascertained that Egr-1 manifestation in CD4^+^ T cells was adversely linked to the potency of EAE in mice. Egr-1-specific eradication in CD4^+^ T cells augmented the EAE severity. Corroboratively, CD4^+^ T cells from MS patients and a DSS-induced IBD mouse model exhibited notably decreased Egr-1 manifestation. These outcomes propose that Egr-1’s presence within CD4^+^ T cells offers protective benefits against self-antigen reactive or inflammatory ailments. The stability between T_H_17 and T_reg_ cells is essential for sustaining immune homeostasis in various self-antigen reactive conditions [23]. An imbalance in T_H_17 and T_reg_ within CD4^+^ T cells, characterized by heightened T_H_17 cell influence and impaired T_reg_ cell function, has been discovered in MS patients [24]. Our study observed notably increased T_H_17 cells and decreased T_reg_ in the spleen, lymph nodes, and CNS of Egr-1 CKO EAE mice compared to CWT EAE mice, implying that T_H_17/T_reg_ imbalance may be the primary cause of exacerbated EAE in CKO mice. However, in vitro differentiation experiments revealed that Egr-1 knockout considerably hampered T_reg_ cell differentiation, while T_H_17 differentiation ability remained unchanged, suggesting that Egr-1 directly regulated T_reg_ cell differentiation rather than T_H_17 cells. The increase in T_H_17 cells in Egr-1 knockout EAE mice may be attributed to Egr-1 indirectly influencing T_H_17 cell ratios by modulating T_reg_ cell differentiation and function, as T_reg_ cells primarily inhibit T_H_17 and other inflammatory cell differentiation and expansion during EAE. Further investigation confirmed Egr-1’s influence on T_reg_ cell suppressor functions, with Egr-1 knockout weakening T_reg_-mediated suppression of conventional T (T_conv_) cells. Thus, Egr-1 in CD4^+^ T cells can regulate T_reg_ cell differentiation and immunosuppressive functions.

T_reg_ cells, distinguished by Foxp3 manifestation, are crucial in treating neuroimmune inflammatory diseases [25]. Investigating T_reg_ cell differentiation primarily focuses on understanding Foxp3 manifestation regulation. TGF-β is widely recognized as a stimulus for Foxp3^+^ T_reg_ cell differentiation in vitro [26]. TGF-β’s intensificational impact on T_reg_ cells relies mainly on up-regulating Foxp3 manifestation via the Smad signaling route. Smad3 enhances Foxp3 manifestation by connection to the CNS1 region of Foxp3 and forming complexes with NFATc2 and CREB that attach to the Foxp3 stimulator region. Additionally, Foxp3 manifestation decreases upon Smad2 deletion [27,28]. Our experiments demonstrated Egr-1’s intensification of endogenous TGF-β production, and previous research showed Egr-1 connection to the TGF-β stimulator, regulating its manifestation. We also discovered a new impact for Egr-1, which enhances Foxp3 manifestation and T_reg_ cell differentiation in vitro independently of TGF-β, indicating that CD4^+^ T lymphocyte Egr-1 directly regulates Foxp3 manifestation rather than indirectly. Notably, our investigation of upstream Egr-1 regulative modulators during T_reg_ differentiation revealed TGF-β’s intensification of T_reg_ cell differentiation by activating the Erk/Egr-1 signaling route. This suggests that TGF-β can induce T_reg_ cell differentiation by activating Raf/Egr-1 in addition to the classical Smad signaling route, emphasizing Egr-1’s importance in T_reg_ differentiation. Our data showed that Smad3 inhibition led to a compensatory up-regulation of Raf/Egr-1 signaling during T_reg_ differentiation. However, interference with Egr-1 simultaneously reduced T_reg_ cell differentiation to a lower level, while Egr-1 overmanifestation compensated for the decreased T_reg_ ratio caused by Smad3 inhibition. These findings indicate that Egr-1 is regulated by the upstream Raf/Erk signaling route, distinct from the classical Smad3 route, and Egr-1 directly regulates Foxp3 manifestation, presenting an alternative novel route for T_reg_ cell differentiation.

Research has demonstrated the critical impact of TCR signaling in Foxp3 manifestation, with induced Foxp3 manifestation observed following TCR signal activation [29]. When stimulated by TCR-CD28 dual signaling, activated downstream genetic modulation modulators such as nuclear factor κB (NF-κB), NFAT, and AP-1 are crucial for inducing Foxp3 manifestation and driving T_reg_ cell differentiation. NFAT, AP-1, Foxo1, and Foxo3 have been shown to bind directly to the Foxp3 stimulator region, regulating Foxp3 genetic modulation [30–32]. Similarly, the genetic modulation modulator Egr-1 was also activated under TCR signal stimulation [33]. Our investigation established that the activated genetic modulation modulator Egr-1 enhances Foxp3 manifestation by connection directly to the Foxp3 stimulator region, consequently promoting T_reg_ cell differentiation. Upon further examination of Egr-1’s impact in MS/EAE, we discovered that administering the Egr-1 agonist CAL mitigated EAE severity in WT mice; however, this curative impact was absent in Egr-1 CKO mice. Studies have also shown that CAL ameliorates DSS-induced colitis in mice. In addition, the mainstream clinically available drugs used to treat MS were studied, including GA, FTY-720, IFN-β, and DEX, all of which could improve mouse EAE. Only FTY-720 and GA treatments resulted in an increased ratio of CD4^+^Egr-1^+^ cells within lymph node cells, suggesting that Egr-1 may serve as a promising curative target for MS. Given the significant impact of Egr-1 in CD4^+^ T cells for EAE and colitis treatment, it is plausible that Egr-1 could represent a potential curative target for various self-antigen reactive diseases, warranting further investigation into its clinical applications.

In summary, our data highlight the importance of Egr-1 in CD4^+^ T cells for the differentiation of T_reg_ cells in the development of MS/EAE. In our findings, Egr-1 activated T_reg_ cell differentiation by connection to the Foxp3 promoter region, which is different from the classic Smad3 route. Furthermore, Egr-1 agonist CAL promoted T_reg_ differentiation and reduced EAE/MS immune and inflammatory responses. Thus, Egr-1 may serve as a novel potential drug target for the treatment of self-antigen reactive diseases.

## Materials and Methods

### Mice

ERE-EGFP mice, Egr-1-floxed (Egr-1^f/+^) mice, and CD4-cre mice were provided by Shanghai Model Organisms Center (Shanghai, China). Egr-1^f/f^ mice were self-bred from Egr-1^f/+^ mice. Egr-1^f/f^ CD4-cre^+^ mice were generated by crossing Egr-1^f/f^ with CD4-cre mice. Female mice, 6 to 8 weeks old, were used unless otherwise indicated. Egr-1^f/f^CD4-cre^−^ and Egr-1^f/f^ CD4-cre^+^ littermates were used as CWT mice and CKO mice, respectively. All mice were kept in the Animal Experimental Center of Shanghai University of Traditional Chinese Medicine and were fed and tested according to the regulations of the Animal Protection and Use Committee of Shanghai University of Traditional Chinese Medicine (PZSHUTCM210715003).

### Cell line and incubation

The Jurkat and EL4 cells were cultivated in RPMI 1640 for Jurkat cells and Dulbecco’s modified Eagle’s medium (Gibco, NY, USA) for EL4 cells. The cultures were nourished at 37 °C in a humidified environment imbued with 5% CO_2_.

### Clinical MS patient serum collection

This study encompassed a total of 5 MS patients in remission and 5 healthy contributors. MS patients were enlisted from Huashan Hospital of Fudan University (Shanghai, China). Ethical endorsement was conferred by the Ethics Committee (KY2021-051). Patients were identified as having relapsing-remitting MS in accordance with the McDonald criteria [34] and had not undergone any disease-modifying therapy. Subsequent to the procurement of written informed consent, blood specimens were garnered harnessing heparinized tubes. CD4^+^ cells were isolated from the blood specimens capitalizing on the EasySep Human CD4 Positive Selection Kit (STEMCELL Technologies, Canada). Cells were then galvanized with anti-human CD3 (5 μg/ml) and anti-human CD28 (2 μg/ml) immunoglobulins for 24 h, after which the expression of Egr-1 and Foxp3 was evaluated by dint of flow cytometry and quantitative PCR.

### Reagents

Deactivated *Mycoplasma tuberculosis* H37RA was secured from BD Biosciences (catalog no. 231141, CA, USA). Incomplete Freund’s adjuvant was fetched from Sigma-Aldrich (catalog no. F5506, MO, USA). CAL, astragaloside I (AS I), astragaloside II (AS II), calycosin-7-glucoside (CG), and FN were purchased from Chengdu Desite Biotech Co. Ltd. (Chengdu, China). Gypenoside IX (GP IX), ginsenoside Rg5 (Rg5), protopanaxadiol (PPD), ginsenoside Re (Re), ginsenoside R1 (R1), and protopanaxatriol (PPT) were provided by the Shanghai Research Center for Standardization of Chinese Medicines (Shanghai, China). Fingolimod (FTY-720) was acquired from MedChemExpress. CD3 (catalog no. 16-0032-85) and CD28 (catalog no. 16-0038-85) immunoglobulins were obtained from eBioscience (CA, USA). IL-4 (catalog no. 504108) and IFN-γ (catalog no. 505812) immunoglobulins were sourced from BioLegend (CA, USA). Anti-TGF-β monoclonal antibody (catalog no. 1D11) was procured from R&D Systems (Minneapolis, MN). Fluorochrome-labeled immunoglobulins targeting CD4 (catalog no. 11-0042-85), IL-17 (catalog no. 17-7177-81), and Foxp3 (catalog no. 88-8111-40) were used to characterize immune cell populations, and Egr-1 (catalog no. 72-5774-40) was purchased from eBioscience. DSS (molecular weight: 36,000 to 50,000 Da) was provided by MP Biochemicals (Irvine, CA).

### Plasmids

The gene manifestation vector, pCMV-SPORT6-Egr-1, was incorporated into the pcB6 plasmid (Promega Biotech, Madison, WI). Primers 5′-CCGCTCGAGGTCTTTATAAAGCCAAGCCATCAGTTC and 5′-CCGGTACCTTACCTGGAGTGGCTGGGTG were utilized to amplify a segment of the mouse Foxp3 stimulator, spanning from −1,864 bp to +316 bp, via PCR. The resulting amplicon was integrated into the pGL3–luciferase–reporter vector (Promega Biotech, Madison, WI), yielding the pGL3–Foxp3–stimulator–reporter (Promoter WT) construct. A deletion mutant, lacking the Egr-1 connection site (from −131 bp to −124 bp), was generated using the primers 5′-AACCCCCATTAATCCCTGCAATTATCAGCACACAC and 5′-AACCCCCATTAATCCCTGCAATTATCAGCACACAC in a PCR. Constructs harboring the mutant Foxp3 stimulator versions were designated as pGL3–Foxp3–stimulator–mutation–reporter (Promoter MT). The integrity of all constructs was confirmed via sequencing (Sangon Biotech, Shanghai, China).

### EAE induction

The induction of EAE in murine subjects was executed as per the method detailed previously [35]. The mice were inoculated with an emulsion comprising incomplete Freund’s adjuvant (200 μl), MOG_35–55_ (300 μg), and H37RA (500 μg) at 3 subcutaneous locations on the dorsal surface of the subjects. Putussis toxin (PTX) was administered (200 ng) on the day of activation, followed by an additional dose 48 h later. The evaluation criteria for neurofunctional scoring are enumerated as follows [36]: 0, denoting normal behavior; 0.5, implying drooping of the tail tip; 1.0, indicative of tail paralysis; 2.0, corresponding to paralysis of a single hind limb or enfeeblement of both hind limbs—dilatory working; 3.0, implying paralysis of both hind legs; 4.0, representing limb paralysis; and 5.0, denoting mortality.

### Histopathology

Following a 21-day period of EAE induction, mice were anesthetized prior to receiving intraventricular injections of 4% paraformaldehyde. The spinal cord was excised and sectioned into 3-μm slices, which were subsequently subjected to hematoxylin and eosin (H&E) and luxol fast blue (LFB) staining procedures. Blinded evaluation of inflammatory infiltration and demyelination was performed using previously reported criteria [37].

### Purification of CNS MNCs

The cerebrum and spinal cord were meticulously extricated from mice and manipulated via mechanical homogenization utilizing a syringe plunger and a 100-μm nylon filter (BD Falcon). Thereafter, MNCs were refined harnessing a 30%/70% (v/v) Percoll gradient (Yisheng, Shanghai, China), in compliance with an entrenched methodology.

### Differentiation of T_H_17 and T_reg_ cells

Immature CD4^+^ T cells (CD4^+^/CD44^low^/CD62L^high^) were obtained from the lymph nodes and spleens of healthy C57BL/6 mice utilizing a specialized Naïve CD4^+^ T Cell Isolation Kit (STEMCELL Technologies, 19765, Canada). The activation of T_H_17 cell differentiation necessitated the enrichment of the incubation medium with anti-mouse IL-4 (10 μg/ml) (BioLegend, 504,108, CA, USA), anti-mouse IFN-γ (10 μg/ml) (BioLegend, 505,812), TGF-β1 (3 ng/ml), IL-1β (10 ng/ml), IL-6 (30 ng/ml), and IL-23 (20 ng/ml). Concurrently, the activation of T_reg_ required the supplementation of TGF-β1 (5 ng/ml) and IL-2 (5 ng/ml) to the incubation medium. In experiments evaluating the CAL-induced intensification of T_reg_ cell differentiation, cells were cultivated under T_reg_-inducing conditions with varying concentrations of CAL (10, 20, and 40 μM).

### Suppression assay of T_reg_ cell

To gauge the inhibitory proficiencies of T_reg_ cells, customary T cells (T_conv_; naïve CD4^+^ T cells extracted from normal C57BL/6 mice) were engaged as responder cells. Naïve CD4^+^ T cells culled from CWT mice and CKO mice were brooded for 3 d in T_reg_-inductive circumstances prior to being co-incubated with carboxyfluorescein diacetate succinimidyl ester (CFSE)-labeled T_conv_ cells (2.5 × 10^5^ cells/well) in a 96-well plate (T_conv_:T_reg_ cell ratios of 8:1, 4:1, and 2:1). Subsequent to 4 d of brooding in RPMI 1640, the fluorescence potency of CFSE was scrutinized via flow cytometry.

### Flow cytometry

For CD3 and CD4 staining, MNCs, spleen, and lymph node cells were incubated in anti-CD3–APC (allophycocyanin) (catalog no. 35-0031-80) and cells were incubated with anti-CD4–FITC (fluorescein isothiocyanate) (catalog no. 11-0042-85) immunoglobulins for a duration of 30 min. Following fixation and permeabilization, staining was performed with anti-IFN-γ–PE (phycoerythrin) and anti-IL-17–APC immunoglobulins. T_reg_ cells were stained using a kit, after which anti-Foxp3–PE antibody was applied. All reagents were procured from eBioscience, and flow cytometry was conducted employing Guava Easycyte 8HT.

### Luciferase assays

The pcB6-Egr-1 and pcB6 (empty) plasmids were transfected into EL4 cells (5 × 10^5^ cells/well) by ViaFect Transfection Reagent (catalog no. E4982, Promega, WI, USA). After 6 h, following 24 h of transfection with reporter constructs and either the Promoter WT vector or Promoter MT vector using a transfection reagent, a luciferase reporter assay was conducted. In all reporter assays, cotransfection with the Renilla–luciferase manifestation vector (Promega) was performed. The Dual Luciferase Assay Kit was employed for the luciferase reporter assay, following the methodology described previously. This kit enables the sequential measurement of firefly (FFL) and Renilla (RL) luciferase activities in a single sample, furnishing an ultra-sensitive methodology for identifying intracellular luciferase activity stemming from stimulator or route stimulation in mammalian cell incubation trials [35] (catalog no. E1960, Promega, WI, USA).

Jurkat cells (5 × 10^5^ cells/well) were seeded in 96-well plates, and the cells were cotransfected with ERE luciferase reporter gene plasmid and Renilla luciferase plasmid and treatment with CAL (50 μM), AS I (50 μM), AS II (50 μM), CG (50 μM), FN (50 μM), GP IX (50 μM), Rg5 (50 μM), PPD (50 μM), Re (50 μM), R1 (50 μM), and PPT (50 μM) was introduced into the cells utilizing ViaFect Transfection Reagent, strictly following the manufacturer’s protocol. Following a 24-hour period, a luciferase reporter assay was executed harnessing the Dual Luciferase Assay Kit. To rectify discrepancies in transfection efficiency among specimens, firefly luciferase activity was standardized in relation to the activity of the cotransfected Renilla luciferase. This approach allowed for accurate comparison of stimulator activity by controlling for variations in transfection efficiency.

### ChIP assay

The ChIP process utilized the Simple-ChIP Plus Enzymatic Chromatin IP Kit (catalog no. 9005, Cell Signaling Technology). In brief, CD4^+^ T cells were subjected to fixation with 1% formaldehyde for a duration of 10 min, followed by lysis. DNA fragmentation was accomplished via sonication, and subsequent co-immunoprecipitation was executed with 1 μg of Egr-1, histone H3 (positive control), and normal rabbit IgG (negative control) antibodies over the course of a single night at a temperature of 4 °C. Antibody/protein complexes were aggregated using Protein G Magnetic Beads, and the ensuing purification process involved 3 iterations of cleansing with ChIP buffer. The succeeding proteinase K digestion was pursued by the derivation of DNA with phenol/chloroform and ethanol precipitation. ChIP DNA was subsequently analyzed employing PCR and quantitative immediate reverse transcriptase PCR (qPCR) with Foxp3–stimulator primer sets (sequences detailed in Table S2). This standard protocol facilitated the examination of protein–DNA interactions and elucidated the impact of Egr-1 in the genetic regulation of Foxp3.

### RNA-sequencing analysis

Merely high-quality RNA samples adhering to the succeeding standards were employed for sequencing library construction and a minimum yield of 2 μg. RNA sequencing was administered at Majorbio Bio-pharm Biotechnology Co. Ltd. (Shanghai, China). To ascertain DEGs between 2 disparate specimens, the manifestation level of each transcript was reckoned predicated on the FPKM methodology (fragments per kilobase of exon per million mapped reads is a normalization method in RNA sequencing that quantifies transcript abundance by adjusting for gene length and sequencing depth). The data were scrutinized on the Majorbio Cloud Platform (www.majorbio.com).

### Real-time PCR

Total RNA was retrieved from CD4^+^ T cells employing the Trizol reagent. The mRNA manifestation levels were quantified utilizing a formerly delineated methodology [38]. The PCR primers are indexed in Table S2.

### Western blotting analysis

Jurkat cells were stimulated with TGF-β (10 ng/ml) for 9 h to investigate the agonistic impact of TGF-β on Egr-1 through the Raf/Mek/Erk route. To confirm this impact, Jurkat cells were pretreated with specific route inhibitors: GW5074 (10 μM; Sigma, MO, USA), an inhibitor of Raf, or U0126 (10 μM; Cell Signaling Technology), an inhibitor of Erk and Mek, for 2 h. Subsequently, TGF-β (10 ng/ml) was introduced for a further 9 h of stimulation. Western blotting was performed as standard procedure [35,36]. Primary immunoglobulins against Egr-1 (catalog no. 4523), Raf (catalog no. 9422), p-Mek (catalog no. 9145), p-Erk (catalog no. 4370), Erk (catalog no. 4696), and glyceraldehyde-3-phosphate dehydrogenase (GAPDH) (catalog no. 5174) were purchased from Cell Signaling Technology. Antibodies against Ras (catalog no. 52939), Mek (catalog no. 178876), p-Raf (catalog no. 173539), and Foxp3 (catalog no. 20034) were purchased from Abcam (London, UK).

### Enzyme-linked immunosorbent assay

Consequent to the dispensation of CAL in EAE mice, splenocytes were segregated from splenic tissue, seeded into 48-well plates, and incited with myelin oligodendrocyte glycoprotein (MOG) for 24 h. Thereafter, the cell incubation supernatant was reaped. The concentrations of extracellular FN-γ, IL-17A, and IL-10 in the supernatant were appraised in compliance with the manufacturer’s protocol (eBioscience).

### Statistical analysis

The distinction between a pair of cohorts was scrutinized employing the Student’s *t* test. All contrasts amidst numerous assemblages were dissected utilizing one-way analysis of variance (ANOVA). A *P* value inferior to 0.05 was esteemed as possessing statistical significance.
