# Supplementary material for: Early Growth Response Gene 1 Benefits Autoimmune Disease by Promoting Regulatory T Cell Differentiation as a Regulator of Foxp3
Source: Research (Wash D C). 2025 Apr 15;8:0662. doi: 10.34133/research.0662 (PMC11997311; doi:10.34133/research.0662)
Supplement: Supplementary 1 — Supplementary Methods Figs. S1 to S7 Tables S1 and S2 [file research.0662.f1.zip › Supplemental Materials-10-24(1).docx]

Supplemental Materials

**Methods**

**1.Acute experimental colitis induction**

Acute experimental colitis was induced in ERE-EGFP mice by administering 3.5% DSS in the drinking water. Eight-week-old female ERE-EGFP mice were randomly divided into two groups: control group and DSS group. Mice in the normal control group drank normal water every day, and mice in the DSS group drank water supplemented with 3.5% DSS for 9 consecutive days, and each mouse was weighed every other day.

**2.Histopathology**

On the 9th day after DSS induction, all mice were anesthetized with pentobarbital sodium and euthanized by cervical dislocation. The entire colon was removed and the length of the colon was measured. The distal colon was then fixed with 4% paraformaldehyde and embedded in paraffin. The tissue sections were stained with HE and the degree of inflammation was scored.**^(1)^**

**Figures and tables**

**Table S1. Statistical summary of clinical features in CWT (Egr-1^f/f^) and CKO (Egr-1^f/f^ CD4-cre^+^) EAE mice.**

| Group | Incidence（%） | Day of onset | Peak score |
| --- | --- | --- | --- |
| CWT EAE | 100 | 10.09 ± 0.3149 | 3.091 ± 0.2506 |
| CKO EAE | 100 | 7.818 ± 0.3770*** | 4.182 ± 0.2635 ** |

n=11; vs CWT EAE mice; **P < 0.01, ***, P < 0.001; mean ± SEM.

**Table S2. Sequences of primers for real-time PCR analysis.**

| Gene | Forward primer | Reverse primer |
| --- | --- | --- |
| Mouse RORγt  Mouse Foxp3  Mouse T-bet  Mouse Egr-1  Mouse GAPDH  Human Foxp3  Human Egr-1  Human GAPDH | GGAAACCAGGCATCCTGAAC  AGGAAAGACAGCAACCTTTTGG  AGCAAGGACGGCGAATGTT  TCCCAGCTCATCAAACCCA  GGGCATCTTGGGCTACACT  TGGCTAGGA AAATGGCA  GGAGACCAGTTACCCCAGCCAAA  AGATGCTACTGGCCGCTGAA | GCACTGCAGAAACTGGGAATG  GGCCACTTGCAGACTCCATT  GGGTGGACATATAAGCGGTTC  GGCAAACTTCCTCCCACAAA  GCCGAGTTGGGATAGGG  GCAGGAGCCCTTGTCGG  TGGAGATGGTGCTGAGGACGAGGAG  TGAAGGTCGGAGTCAACGGATTTGGT |


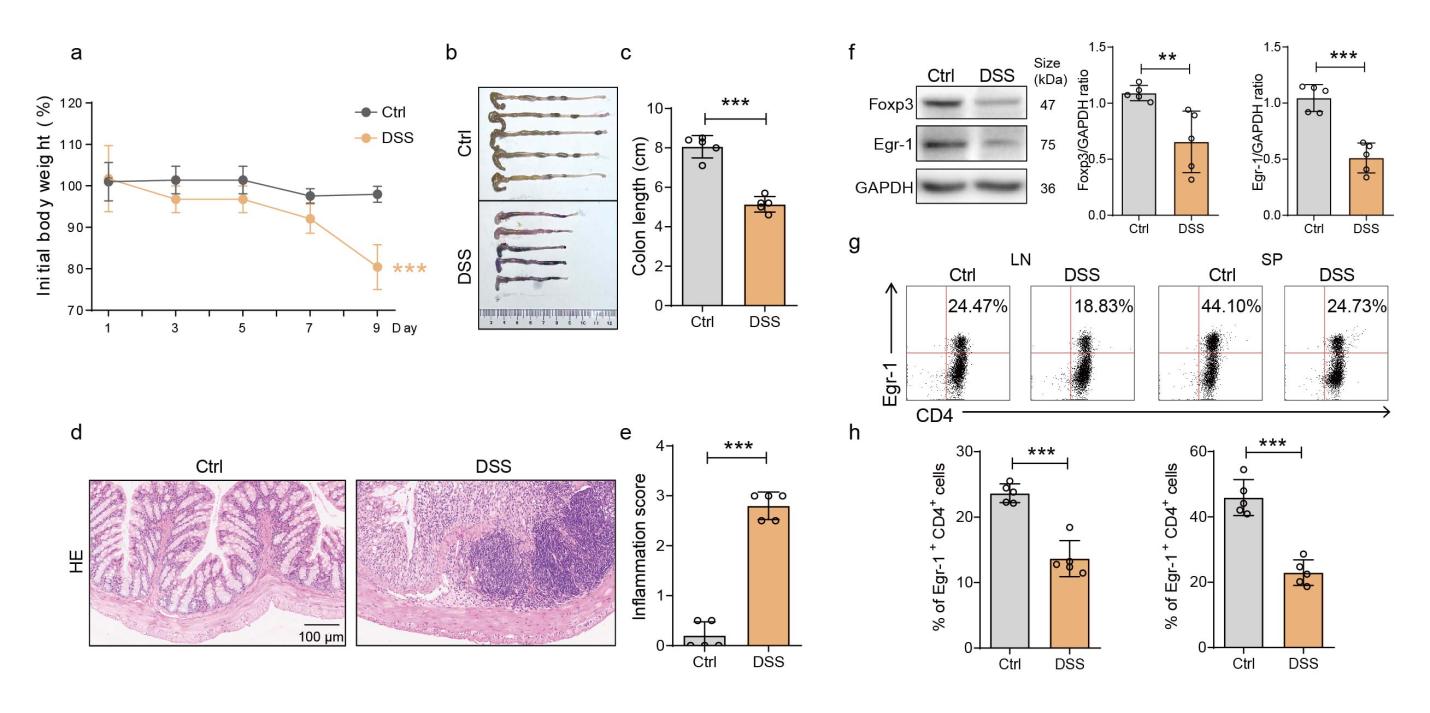


Figure S1. **Egr-1 expression was down-regulated in DSS-induced colitis mice. a** Percentage of body weight of mice compared to day 1. **b-c** Colon and length statistics of two groups of mice. **d-e** HE staining and inflammatory infiltration score of colon tissue. **f** Expression of Foxp3 and Egr-1 in colon tissue. **g-h** Proportion of Egr-1^+^ cells in spleen and mesenteric lymph nodes of mice. n=5. Data are expressed as mean ± SD. **, P < 0.01; ***, P < 0.001 by student’s t test.


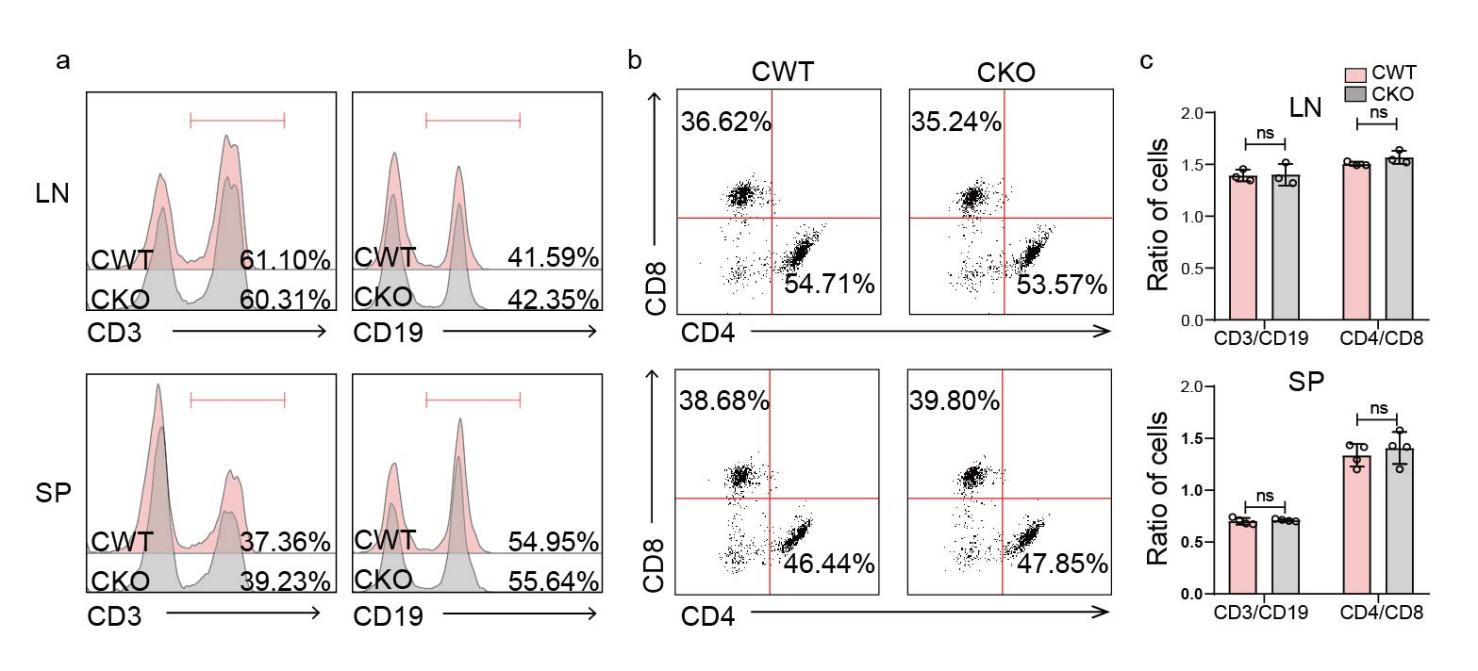


Figure S2. **Proportion of immune cells in CWT/CKO mice. a** Proportion of CD3 and CD19 cells in lymph nodes and spleen. **b** Proportion of CD8 and CD4 cells in lymph nodes and spleen. **c** Ratio of CD3/CD19 and CD4/CD8 in CWT or CKO mice. n=3-4. Data are expressed as mean ± SD. ns, P > 0.05 by student’s t test.


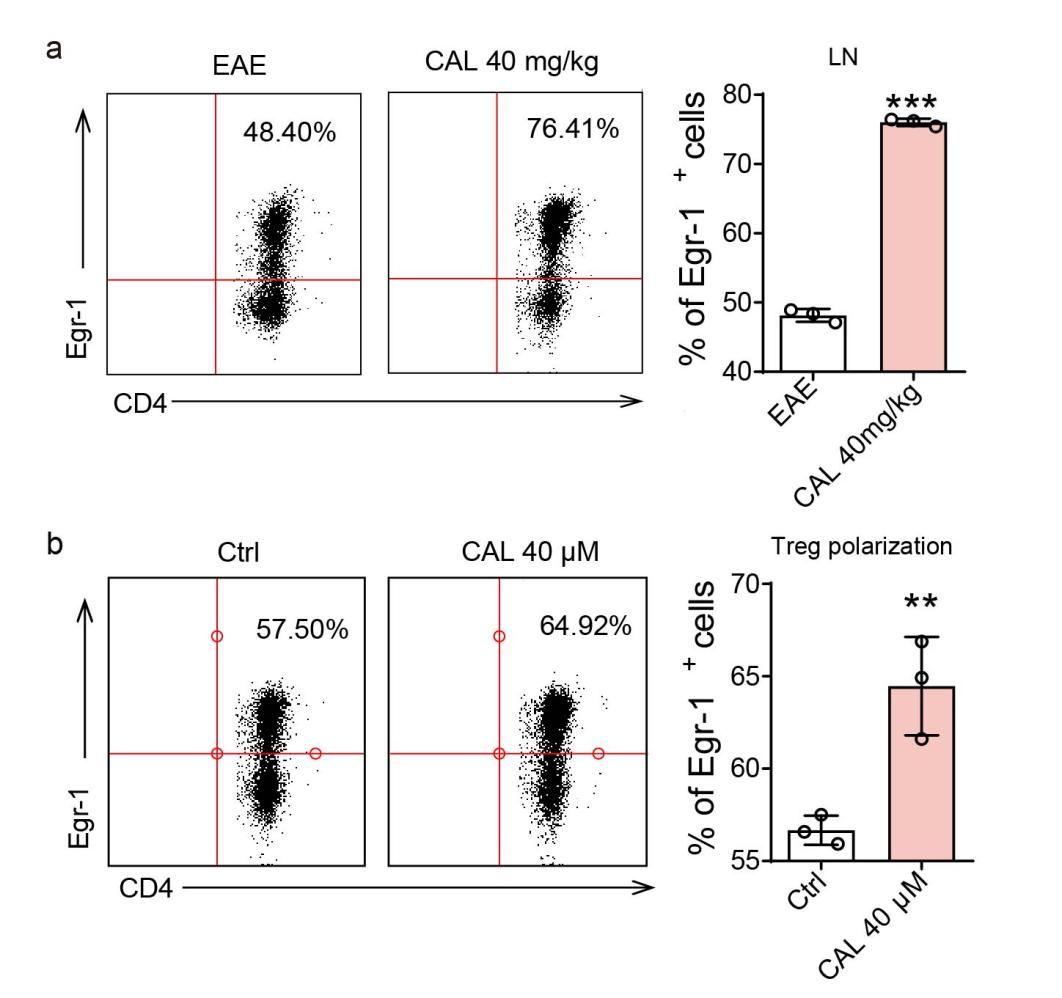


Figure S3. **CAL activated Egr-1 expression both *in vivo* and *in vitro.*** **a** ERE-EGFP mice were given CAL from day 9 to day 21 after EAE induction, and the proportion of CD^+^Egr-1^+^ cells in lymph nodes was detected. **b** CAL was added during Treg polarization to detect the proportion of CD^+^Egr-1^+^ cells. n=3. Data are expressed as mean ± SD. **, P < 0.01; ***, P < 0.001 by student’s t test.


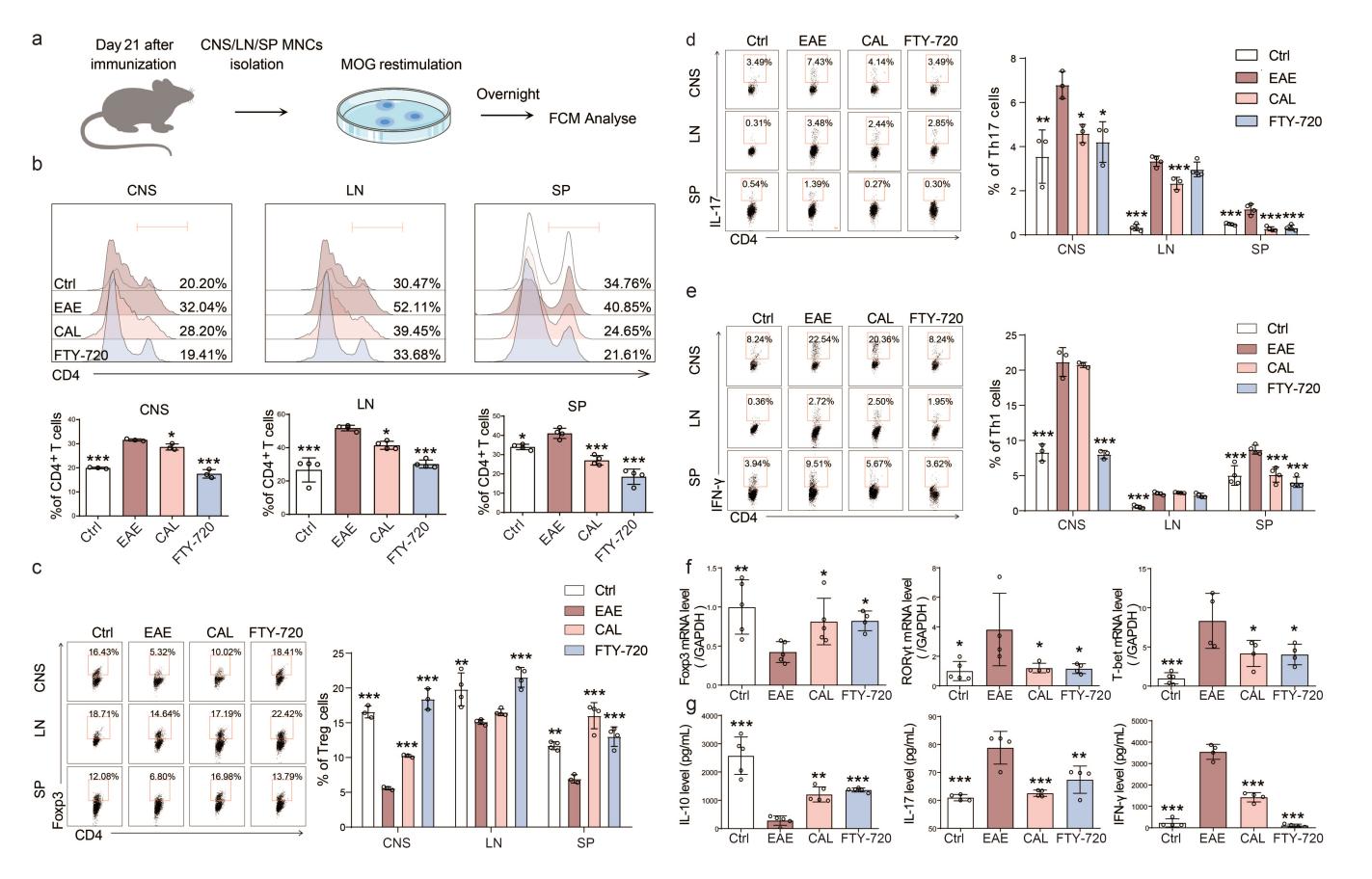


**Figure S4.** **CAL regulated the proportion of CD4^+^ cells and differentiation of CD4 subtypes in EAE mice.** **a-b** Proportion of CD4^+^ cells in CNS, spleen (SP) and lymph nodes (LN) of mice on day 21 after immunization. n=3-4. **c** Proportion of CD4^+^Foxp3^+^Treg cells in CNS, SP and LN of mice. **d** Proportion of CD4^+^IL-17^+^Th17 cells in CNS, SP and LN of mice. **e** Proportion of CD4^+^IFN-γ^+^Th1 cells in CNS, SP and LN of mice. n=3-4. **f** Expressions of Foxp3, RORγT and T-bet mRNA and **g** secretion levels of IL-10, IL-17 and IFN-γ in spleen of EAE mice. n=4-5. Data are expressed as mean ± SD. vs. EAE group, **, P < 0.01; ***, P < 0.001 by one-way ANOVA with Dunnett's multiple comparisons test.


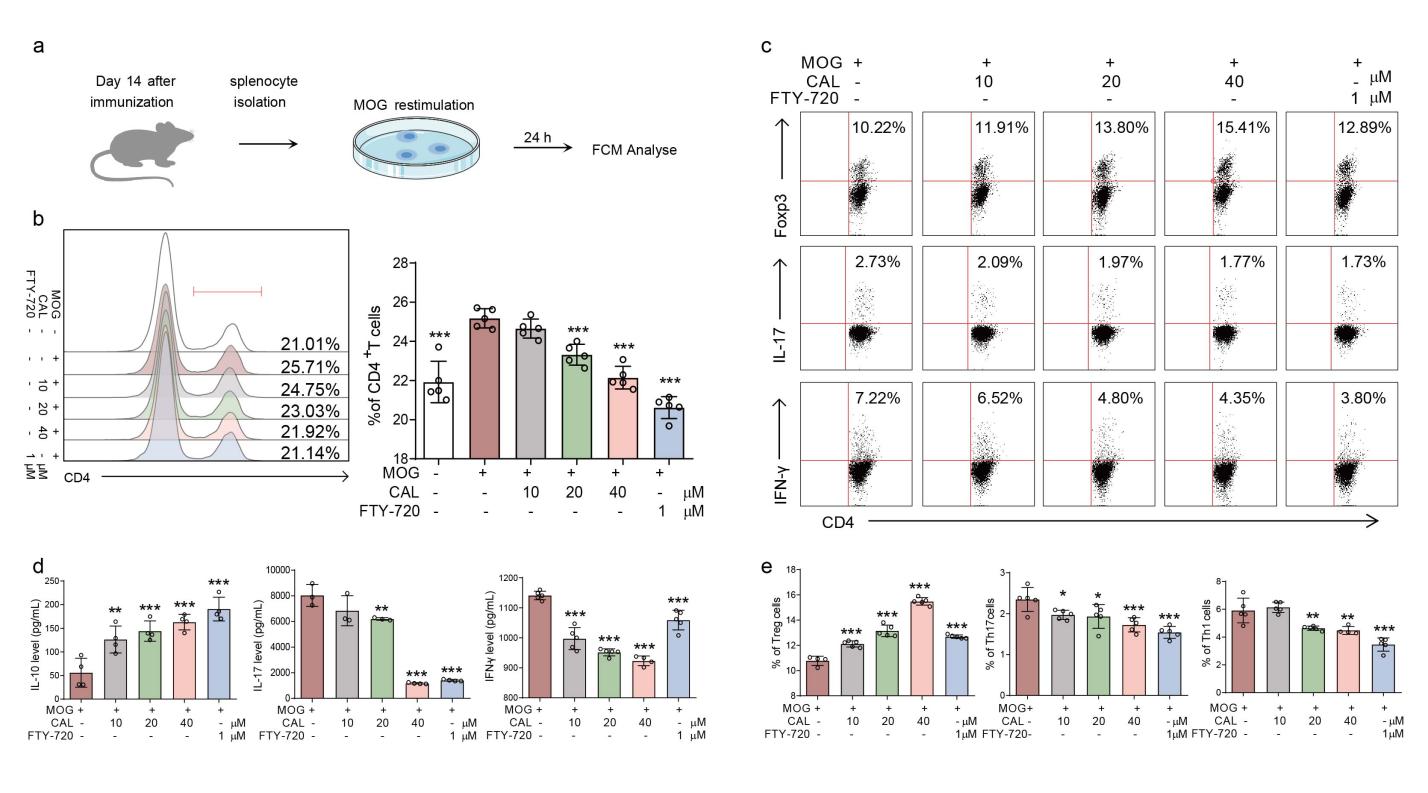


**Figure S5.** **CAL regulated MOG-induced CD4^+^T cell proportion and CD4 subtype differentiation *in vitro*. a** On the 14th day after immunization of EAE mice, spleen tissue was ground into single-cell suspension and cultured for 24 h after adding MOG and CAL or FTY-720. **b** Proportion of CD4^+^ cells. n=5. **c, e** Proportion of CD4^+^Foxp3^+^Treg cells, CD4^+^IL-17^+^Th17 cells, CD4^+^IFN-γ^+^Th1 cells. n=4-5. **d** Secretion levels of IL-10, IL-17 and IFN-γ. n=4-5. Data are expressed as mean ± SD. vs. MOG group, **, P < 0.01; ***, P < 0.001 by one-way ANOVA with Dunnett's multiple comparisons test.


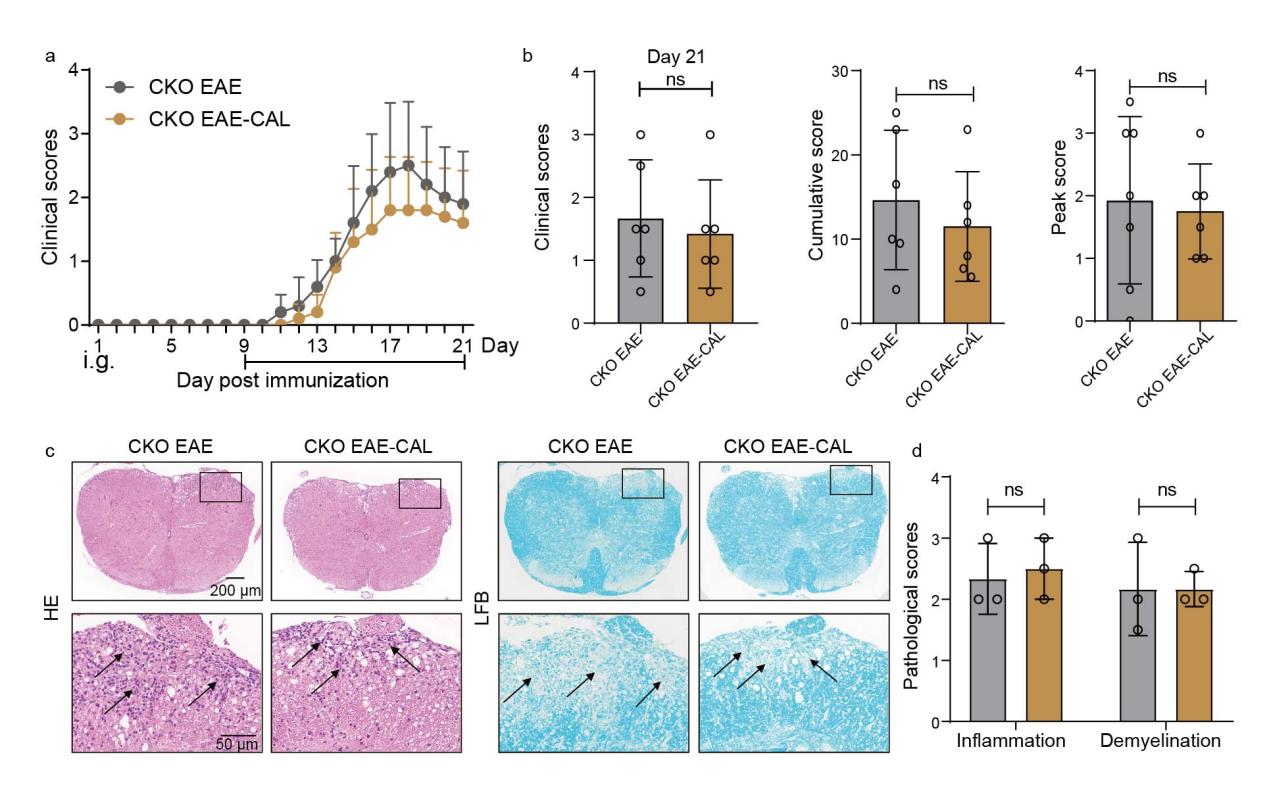


**Figure S6 Therapeutic effect of CAL on EAE was dependent on CD4^+^Egr-1. a Daily neurobehavioral assessment of CKO EAE mice treated with CAL or vehicle.** n=6. **b** Cumulative score, peak score and neurobehavioral assessment on day 21 of EAE mice. n=6. **c-d** HE and LFB staining and inflammatory infiltration and demyelination scores in the spinal cord of EAE mice. n=3. Data are expressed as mean ± SD. ns, P > 0.05; *, P < 0.05; **, P < 0.01; ***, P < 0.001 by student’s t test.


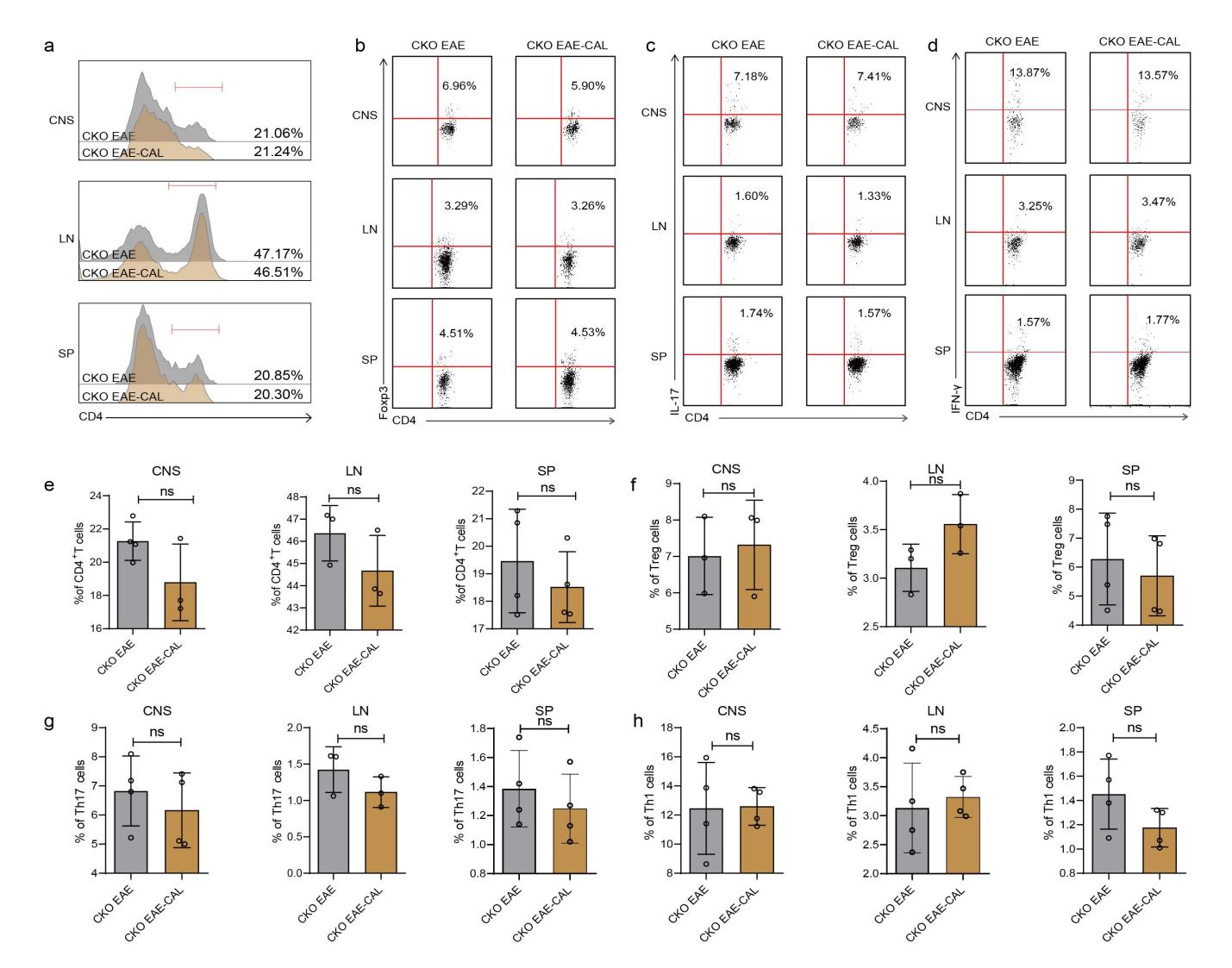


**Figure S7.** **CAL had no effect on CD4 differentiation in CKO EAE mice. a, e** Proportion of CD4^+^ cells in CNS, SP and LN of mice on day 21 after immunization. **b-d, f-h** Proportion of CD4^+^Foxp3^+^Treg cells, CD4^+^IL-17^+^Th17 cells, CD4^+^IFN-γ^+^Th1 cells in CNS, SP and LN of mice. n=3-4. Data are expressed as mean ± SD. **, P < 0.01; ***, P < 0.001 by student’s t test.

References

1.Yue B. and Ren J.Y., et al. Pinocembrin alleviates ulcerative colitis in mice via regulating gut microbiota, suppressing TLR4/MD2/NF-κB pathway and promoting intestinal barrier. Biosci Rep. 2020, 40(7):BSR20200986.
