# Supplementary figures and images for: Early Growth Response Gene 1 Benefits Autoimmune Disease by Promoting Regulatory T Cell Differentiation as a Regulator of Foxp3
Source: Research (Wash D C). 2025 Apr 15;8:0662. doi: 10.34133/research.0662 (PMC11997311; doi:10.34133/research.0662)

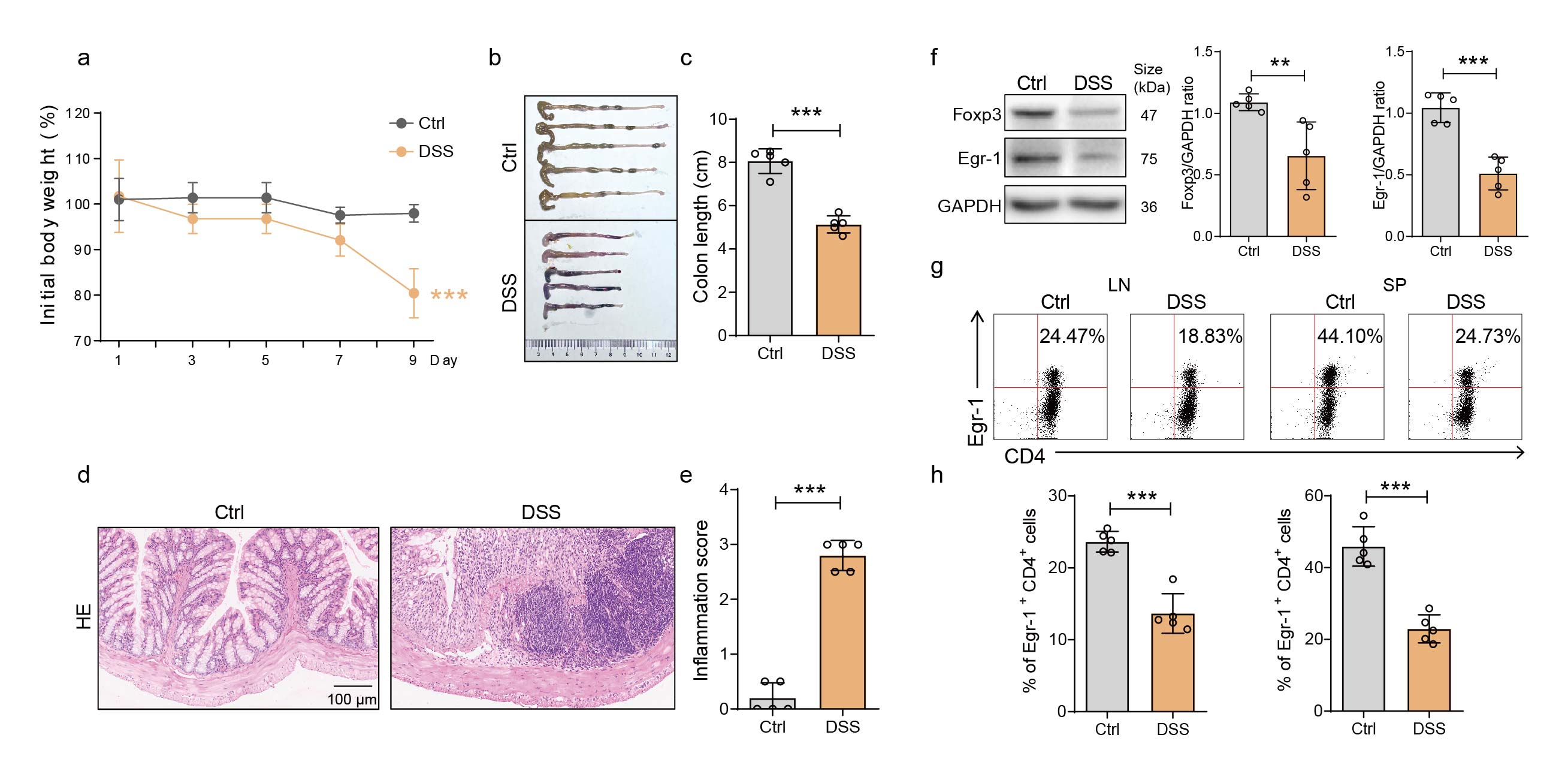

Supplement: Supplementary 1 — Supplementary Methods Figs. S1 to S7 Tables S1 and S2 [file research.0662.f1.zip › figS1-01.jpg]

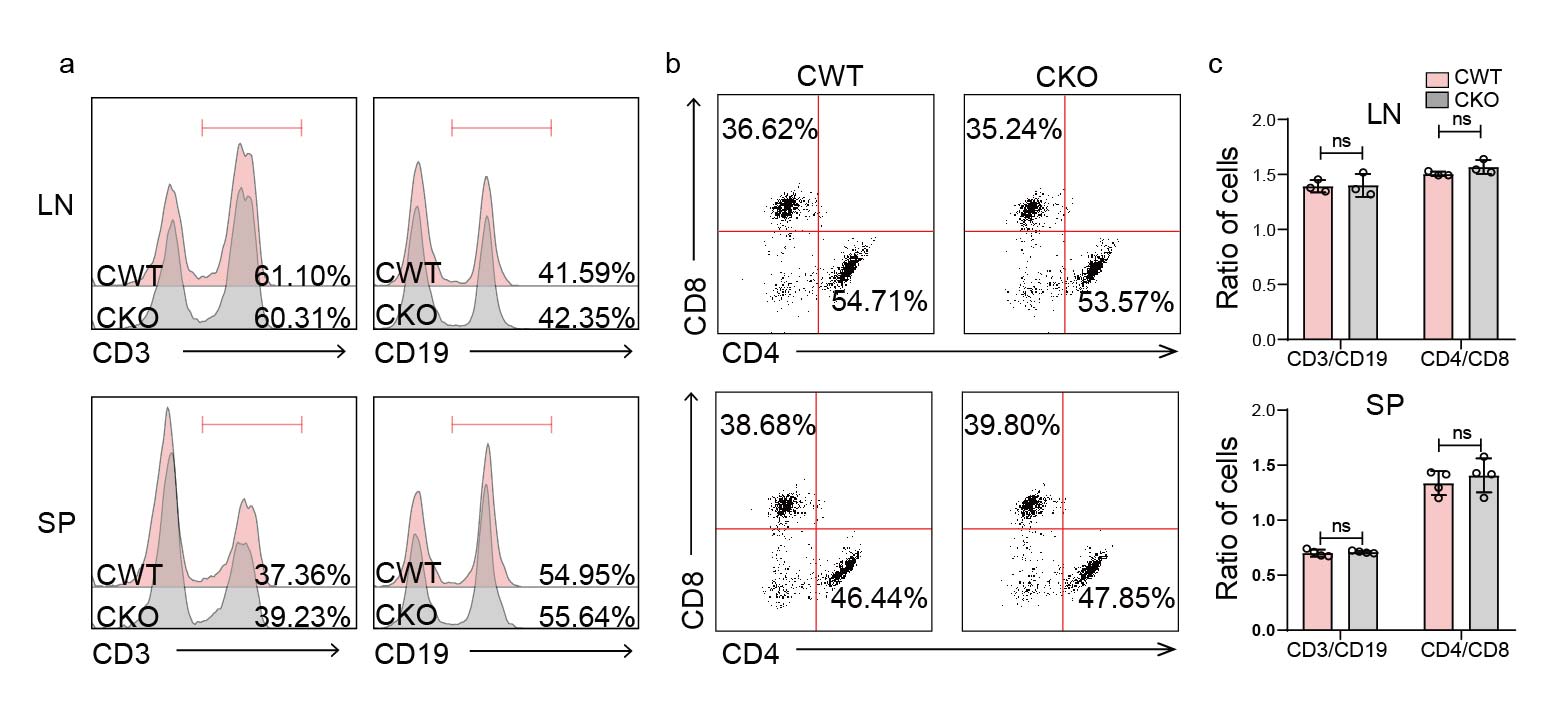

Supplement: Supplementary 1 — Supplementary Methods Figs. S1 to S7 Tables S1 and S2 [file research.0662.f1.zip › figS2-01.jpg]

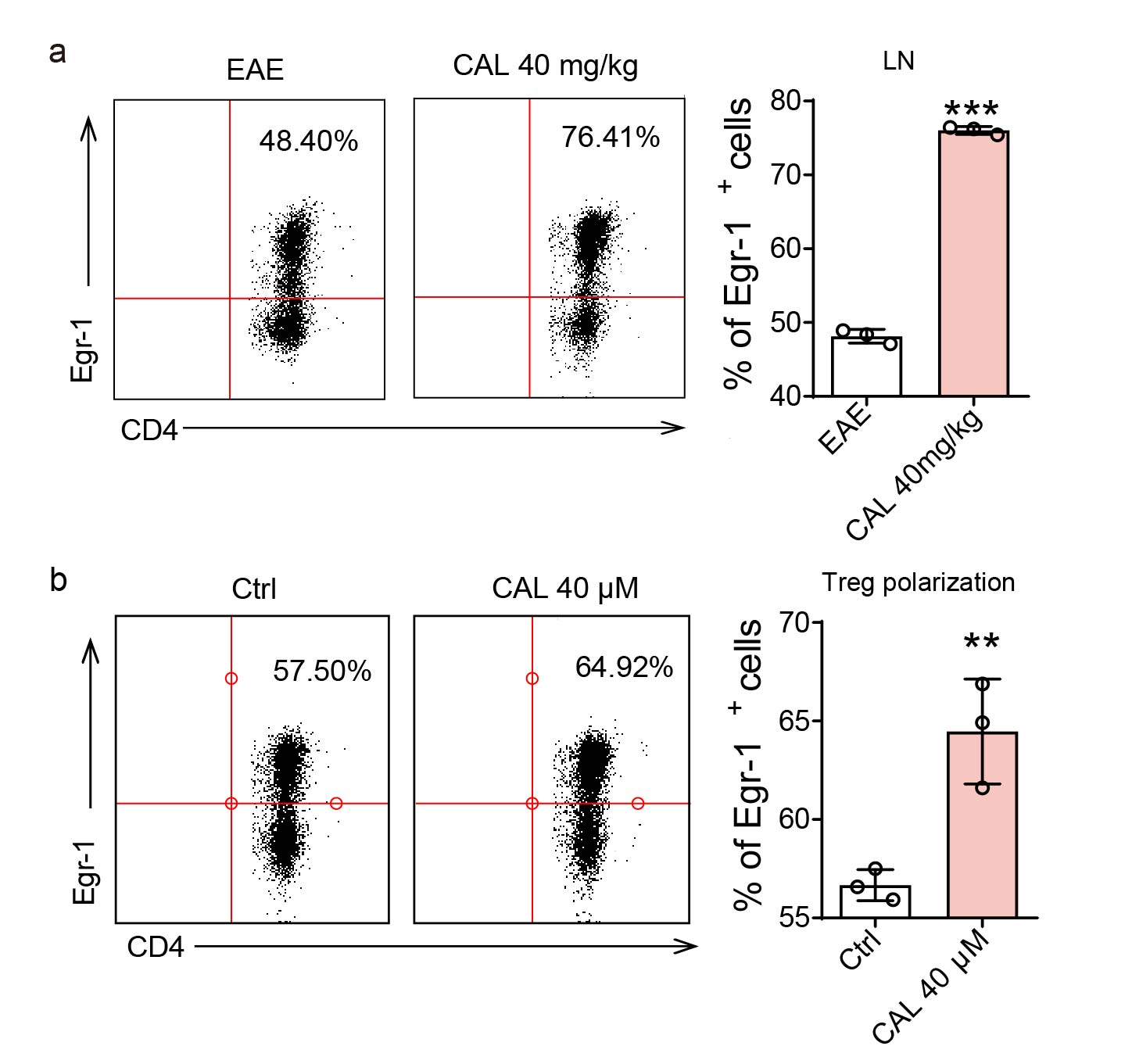

Supplement: Supplementary 1 — Supplementary Methods Figs. S1 to S7 Tables S1 and S2 [file research.0662.f1.zip › figS3.jpg]

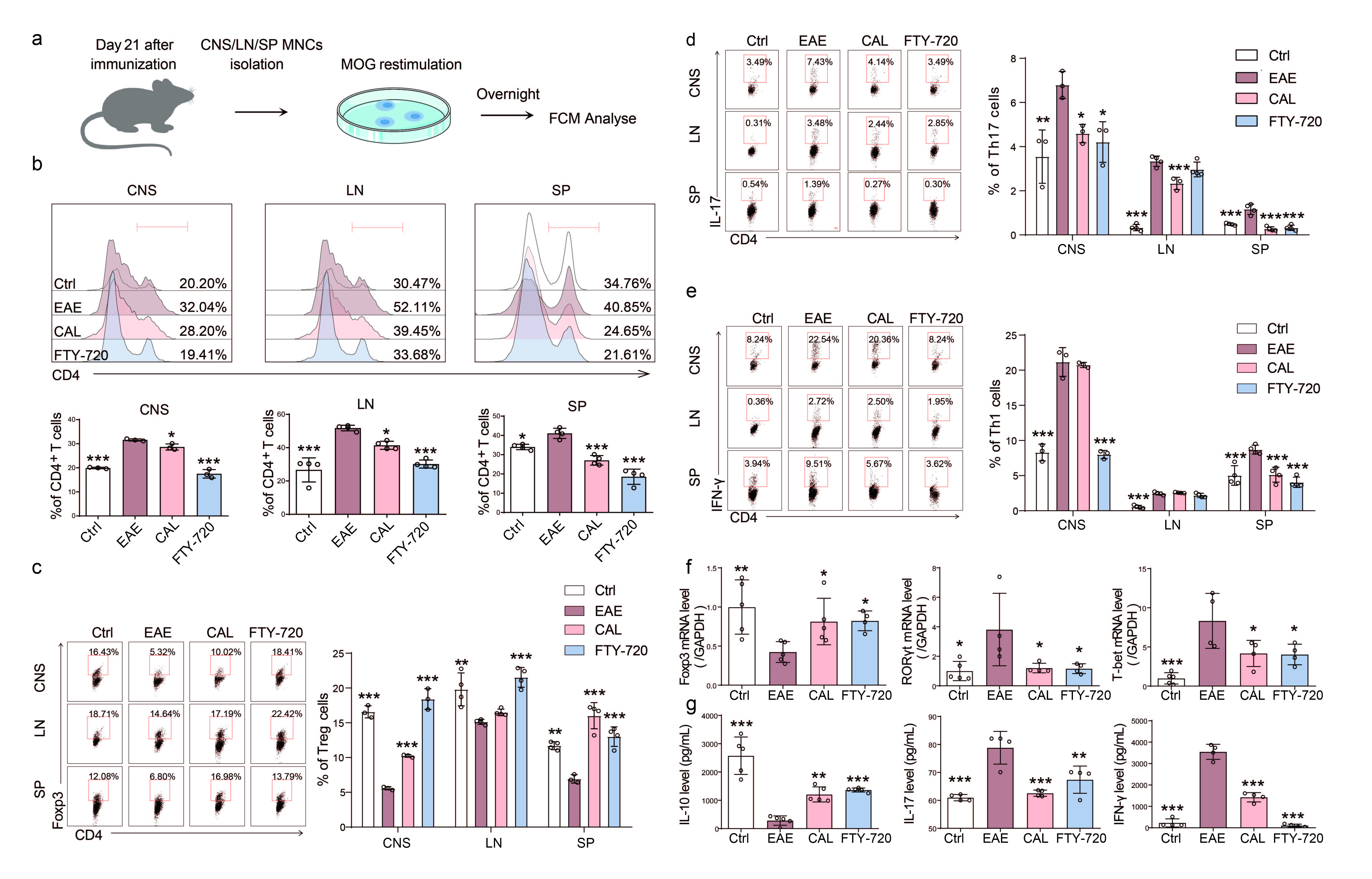

Supplement: Supplementary 1 — Supplementary Methods Figs. S1 to S7 Tables S1 and S2 [file research.0662.f1.zip › figS4.jpg]

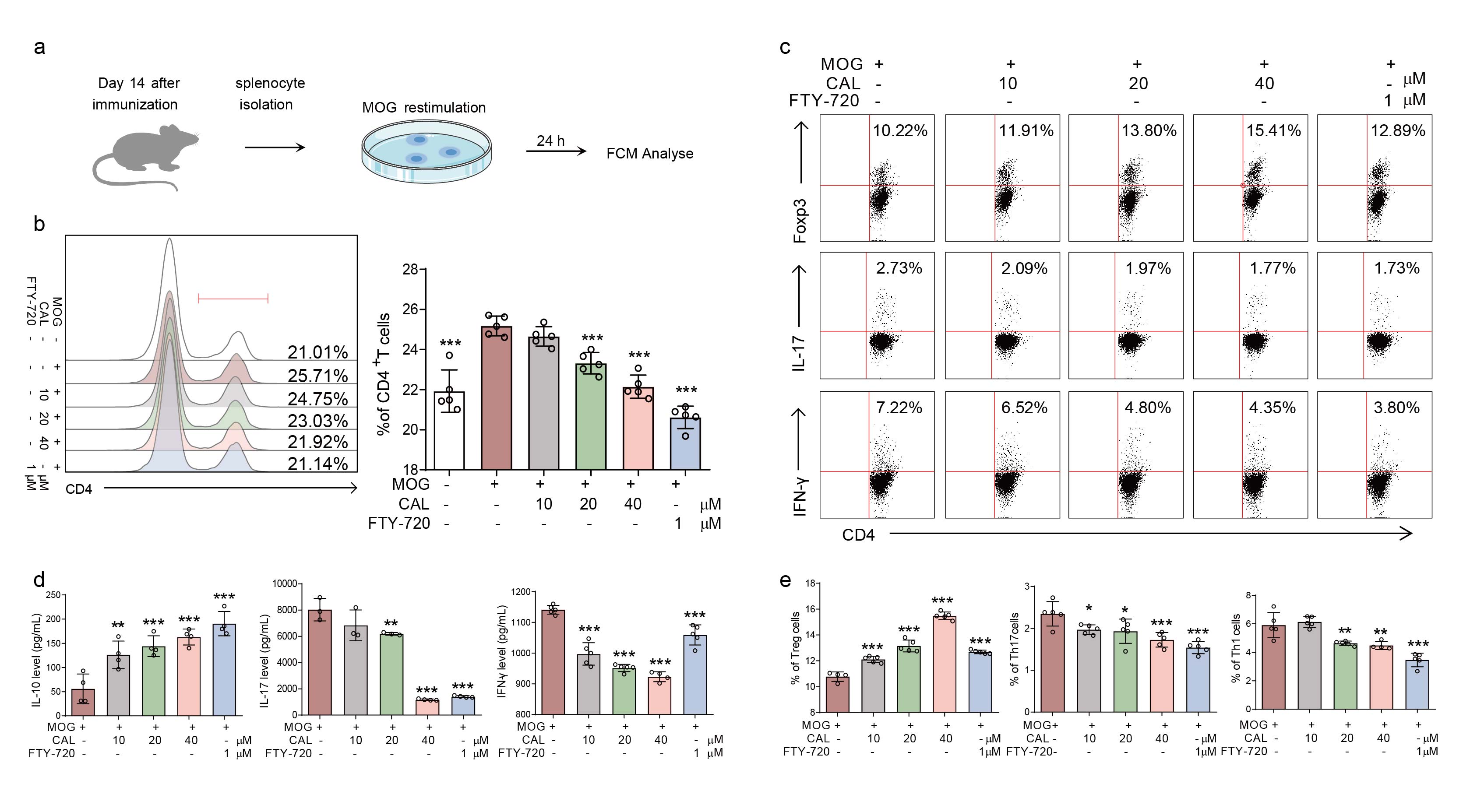

Supplement: Supplementary 1 — Supplementary Methods Figs. S1 to S7 Tables S1 and S2 [file research.0662.f1.zip › figS5.jpg]

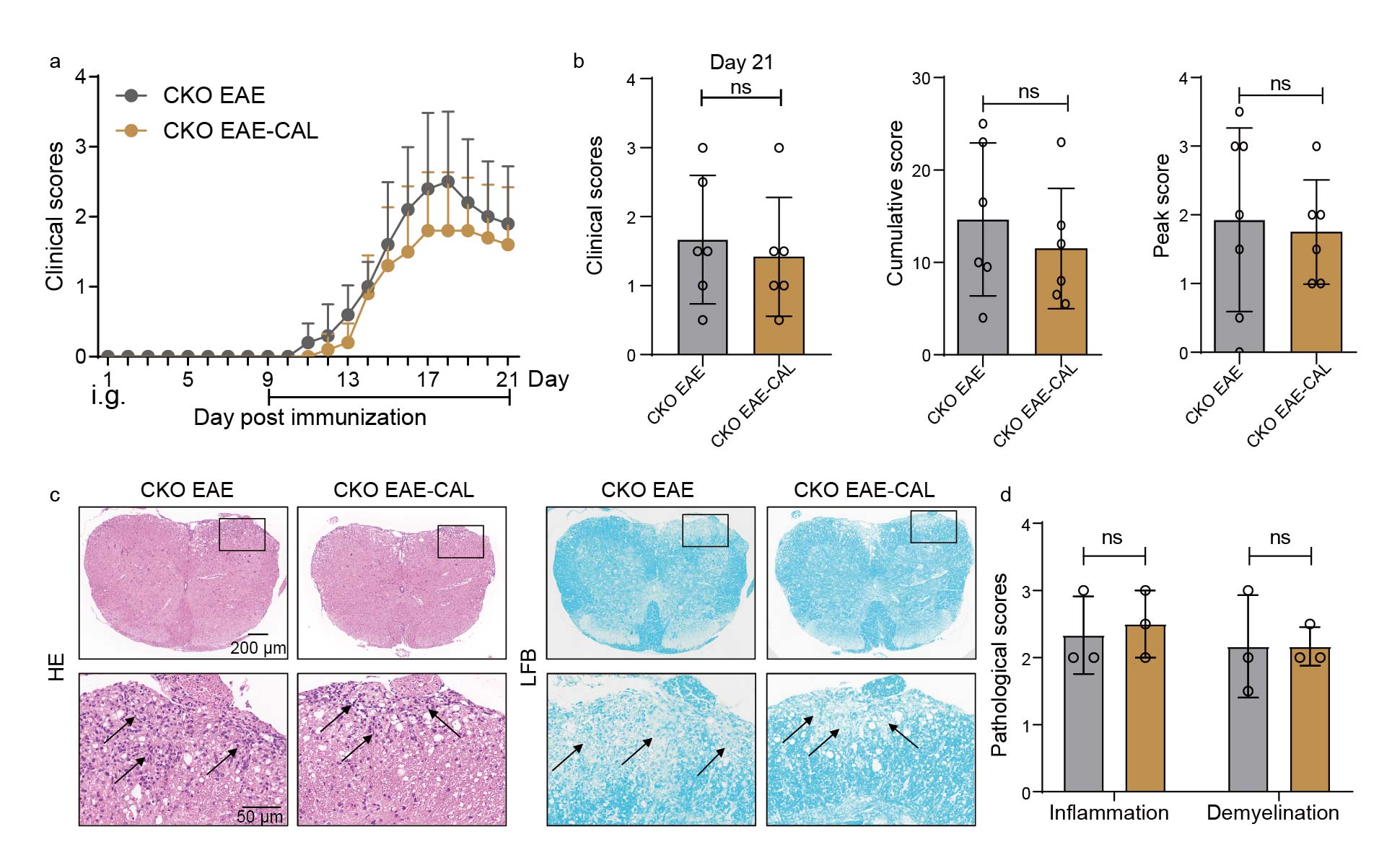

Supplement: Supplementary 1 — Supplementary Methods Figs. S1 to S7 Tables S1 and S2 [file research.0662.f1.zip › figS6.jpg]

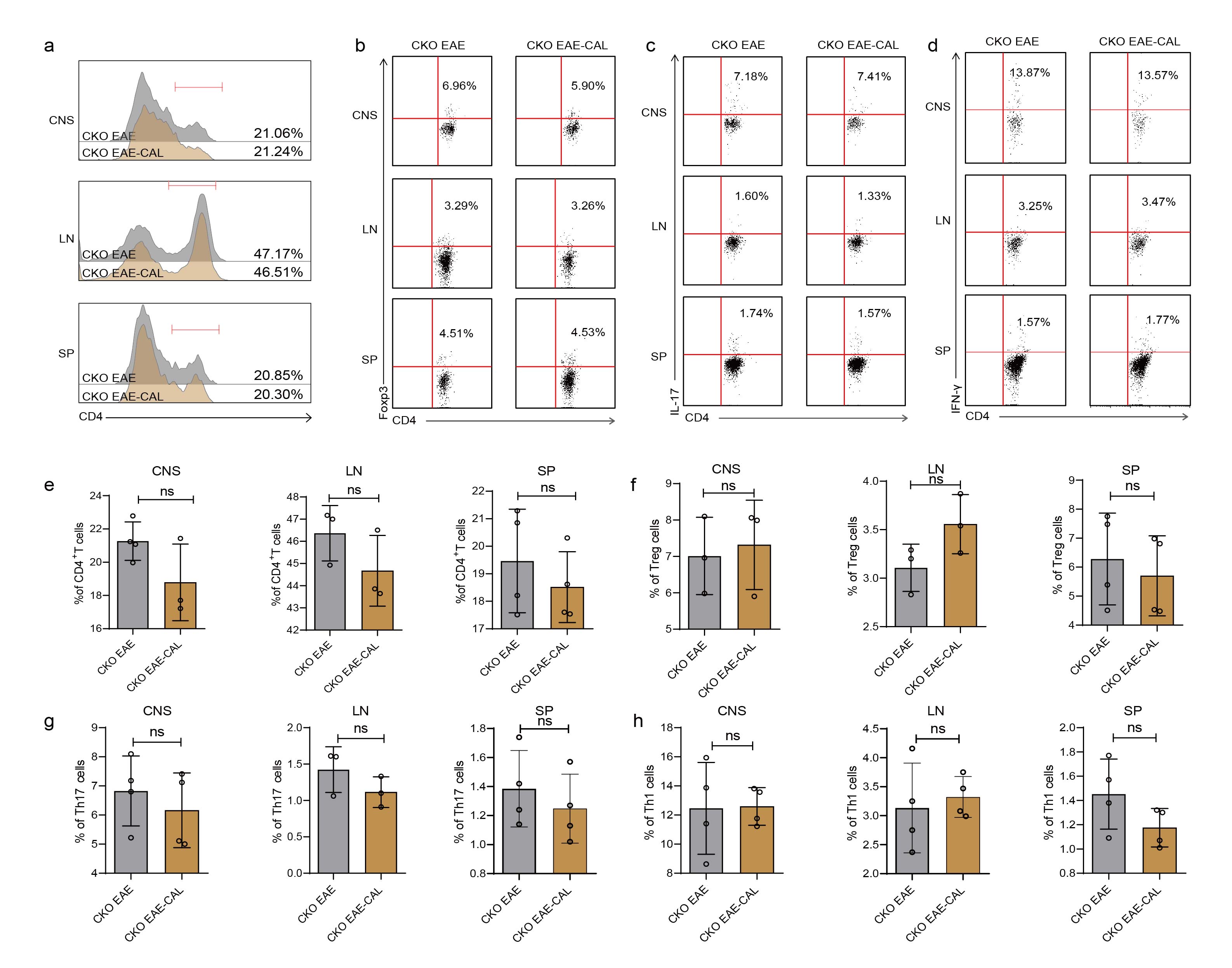

Supplement: Supplementary 1 — Supplementary Methods Figs. S1 to S7 Tables S1 and S2 [file research.0662.f1.zip › figS7.jpg]
